# Invention of 3Mint for feature grouping and scoring in multi-omics

**DOI:** 10.3389/fgene.2023.1093326

**Published:** 2023-03-15

**Authors:** Miray Unlu Yazici, J. S. Marron, Burcu Bakir-Gungor, Fei Zou, Malik Yousef

**Affiliations:** ^1^ Department of Bioengineering, Abdullah Gül University, Kayseri, Türkiye; ^2^ Department of Statistics and Operations Research, University of North Carolina, Chapel Hill, NC, United States; ^3^ Department of Computer Engineering, Abdullah Gul University, Kayseri, Türkiye; ^4^ Department of Biostatistics, University of North Carolina at Chapel Hill, Chapel Hill, NC, United States; ^5^ Department of Genetics, University of North Carolina at Chapel Hill, Chapel Hill, NC, United States; ^6^ Department of Information Systems, Zefat Academic College, Zefat, Israel; ^7^ Galilee Digital Health Research Center, Zefat Academic College, Zefat, Israel

**Keywords:** multi-omics, machine learning, breast cancer, integrative analysis, miRNA

## Abstract

Advanced genomic and molecular profiling technologies accelerated the enlightenment of the regulatory mechanisms behind cancer development and progression, and the targeted therapies in patients. Along this line, intense studies with immense amounts of biological information have boosted the discovery of molecular biomarkers. Cancer is one of the leading causes of death around the world in recent years. Elucidation of genomic and epigenetic factors in Breast Cancer (BRCA) can provide a roadmap to uncover the disease mechanisms. Accordingly, unraveling the possible systematic connections between-omics data types and their contribution to BRCA tumor progression is crucial. In this study, we have developed a novel machine learning (ML) based integrative approach for multi-omics data analysis. This integrative approach combines information from gene expression (mRNA), microRNA (miRNA) and methylation data. Due to the complexity of cancer, this integrated data is expected to improve the prediction, diagnosis and treatment of disease through patterns only available from the 3-way interactions between these 3-omics datasets. In addition, the proposed method bridges the interpretation gap between the disease mechanisms that drive onset and progression. Our fundamental contribution is the 3 Multi-omics integrative tool (3Mint). This tool aims to perform grouping and scoring of groups using biological knowledge. Another major goal is improved gene selection *via* detection of novel groups of cross-omics biomarkers. Performance of 3Mint is assessed using different metrics. Our computational performance evaluations showed that the 3Mint classifies the BRCA molecular subtypes with lower number of genes when compared to the miRcorrNet tool which uses miRNA and mRNA gene expression profiles in terms of similar performance metrics (95% Accuracy). The incorporation of methylation data in 3Mint yields a much more focused analysis. The 3Mint tool and all other supplementary files are available at https://github.com/malikyousef/3Mint/.

## 1 Introduction

During the last 2 decades, the elucidation and comprehensive understanding of complex biological processes in disease development have been enhanced with the advent of sequencing technology. Immense amounts of biological information generated with these technologies; and promising tools and methods have enabled the integration and interpretation of molecular mechanisms of complex diseases at different levels. Availability of multi-omics data such as genomics, transcriptomics, proteomics, epigenomics data helps in bridging the gap between disease onset and progression mechanisms and complex roles of biomolecules (Bersanelli et al., 2016; Yan et al., 2017). Integration of multi-omics data also facilitates the identification of disease subtypes and biomarker prediction.

Cancer defined as a complex disease with uncontrolled cell proliferation is one of the leading causes of death worldwide. According to the World Health Organization statistics in 2020 one out of six deaths, nearly 10 million deaths are associated with cancer (Ferlay et al., 2020). Two most common cancer types are breast cancer (BRCA) with 2.26 million cases, followed by lung cancer with 2.21 million cases. Other most common cancer types are colon, rectum, prostate and skin cancers.

Cancer development is affected by multiple factors such as genetic mutations in genes including oncogenes and tumor suppressor genes and the interaction of inherited factors with various environmental factors, e.g., lifestyle, diet and exposure to carcinogens. Understanding the genomic, epigenomic regulations and enlightening the systematic connections between-omics data types in cancer can provide invaluable information to analyze cellular or intercellular activities and the underlying characteristics of disease mechanisms. The studies have enabled the identification of different biological footprints (biomarkers) derived from different-omics data. For example, HER2 (epidermal growth factor receptor II) overexpression leads to uncontrolled cell growth. HER2+ breast cancer cells having higher levels of HER2 protein tend to be aggressive and grow faster compared to breast cancer cells with low level expression of HER2 (HER2-). Patients with HER2+ BRCA tend to have worse prognosis than patients whose HER2 are not overexpressed (HER2-). As another example, the microRNA hsa-miR-206 decreases the metastatic potential of BRCA cells. Yet as another example, upregulated miRNAs of miR-21 and miR-181 in triple negative BRCAs shorten the overall survival *via* metastatic characteristics (Yan et al., 2008; Neel and Lebrun, 2013). Several studies demonstrated significant hypomethylation of HYAL2 (Hyaluronoglucosaminidase 2) in BRCA cases, hypermethylation of DOK7 (docking protein 7) and KIF1A (kinesin family member 1A) in promoter regions of BRCA samples (Heyn et al., 2013; Guerrero-Preston et al., 2014; Yang et al., 2015). These examples show how methylation level of the associated genes can also be utilized as potential biomarkers for early detection of BRCA. Along this line, various integrative analyses of-omics data have been recently proposed: e.g., integrating DNA methylation and mRNA data (Gong et al., 2021), integrating miRNA expression patterns and DNA methylation (Gong et al., 2021), copy number variations and mRNA expression (Xia et al., 2019). Following that, more-omics data types have been employed to yield a better understanding and more complete picture of the heterogeneous cancer disease, as in (Sun et al., 2018; Tong et al., 2020). However, heterogenous noisy biological data handling is a notoriously challenging job (Fu et al., 2011). Statistical analysis of multi-dimensional data is another rough side of biological data integration because of the “curse of dimensionality” adversity, as initially named by Bellman (1961). Notable feature selection methods have been introduced to reveal distinct molecular patterns for dysregulated cellular mechanisms. These methods are basically divided into unsupervised and supervised data integration methods. While matrix factorization, Bayesian, network-based, and other multivariate approaches are involved in unsupervised methods; network based, multi-kernel and multi-step approaches are collected under the umbrella of supervised methods. Joint Non-negative Matrix Factorization (NMF) (Zhang et al., 2012), iCluster+ (Mo et al., 2013), Multiple Dataset Integration (MDI) (Kirk et al., 2012) and JIVE (Lock et al., 2013) have been proposed as unsupervised methods; and supervised approaches such as Network-based integration of multi-omics data (NetICS) (Dimitrakopoulos et al., 2018), Analysis Tool for Heritable and Environmental Network Associations (ATHENA) (Kim et al., 2013), The Feature Selection Multiple Kernel Learning (FSMKL) (Seoane et al., 2014) compile different-omics data layers to elucidate the biological insight of diseases. According to a previous report, a deep learning-based prediction model is developed to integrate multi-omics data (ElKarami et al., 2022). Gene similarity network (GSN) maps are generated by extracting the feature relationships using the UMAP dimension reduction method. Then, disease types and tumour stages are classified through the use of convolutional neural networks (CNN). A classification method based on prognostic measure is introduced in a recent study (Zhou et al., 2022). The gene similarity network maps created for each-omics data are merged into the residual neural network model. MiBiOmics tool focuses on the identification of the associations across omics datasets and provides robust link detection between-omics layers (Zoppi et al., 2021). This network based approach with Sparse Partial Least Square Discriminant Analysis is used for feature selection, analysis and visualization of biomarker networks in multi-omics data. A multi-omics integrative pipeline, STATegRa, is developed to combine multi-omic analysis tools and provides a systematic approach by using component, non-parametric combination and exploratory analyses (Planell et al., 2021). DeepProg tool provides semi-supervised hybrid computational modeling framework (Poirion et al., 2021). Survival risk of patients is predicted by integrating deep learning and ML models. On the other hand, a study showed that the detection of global signatures and survival subtypes *via* Support vector machine models can provide an informative basis for systematic characterizations in-omics level in pan-cancer analysis (Cheerla and Gevaert, 2017).

Great strides have been made in data analysis approaches with the emergence of RNA-Seq based profiling technology. Most of the previous methods include applications of traditional ML algorithms to transcriptomic data. In these methods, the basis of most feature selection algorithms applied to gene expression data depends on pure statistics and ML (Bellazzi and Zupan, 2007), without considering the underlying biology. The incorporation of biological knowledge into the ML algorithms have shifted the pure data-oriented studies into the domain knowledge driven ML approaches. KEGG pathways (Kanehisa et al., 2017), Gene Ontology (GO) (The Gene Ontology Consortium, 2019), miRNA database (Huang et al., 2019b) are commonly used sources of biological domain knowledge for considering deep analysis of the data. Developing advanced tools based on such given biological knowledge provides a comprehensive framework for complex diseases. To this end, Yousef et al. (2021a) proposed a grouping-based feature selection method [Grouping-Scoring-Modeling (G-S-M)], where the groups can be generated *via* 1) using pre-existing biological knowledge (PBK) stored in a database (such as mirTarBase (Huang et al., 2019b), DisGeNET (Piñero et al., 2019), KEGG PATHWAY [Kanehisa et al., 2017)]; or 2) fully data driven approach using statistical measures such as Pearson correlations. This generic approach has been used by several bioinformatics tools such that some of them use PBK while others are fully data-driven in terms of detecting the groups. An example of such tools are: miRcorrNet (Yousef et al., 2021a) and miRModuleNet (Yousef et al., 2022a), which detect groups from mRNA and miRNA omics datasets; maTE (Yousef et al., 2019) that uses microRNA target genes as the groups; SVM-RNE (Yousef et al., 2009); Integrating of Gene Ontology (Yousef et al., 2021b) that uses Gene Ontology information for grouping; CogNet (Yousef et al., 2021c) and PriPath (Yousef et al., 2022b) that use KEGG pathways for grouping; SVM-RCE (Yousef et al., 2021d) that detects groups by running clustering algorithms such as k-means; TextNetTopics (Yousef and Voskergian, 2022) that uses text topics as groups; GediNet (Yousef and Qumsiyeh, 2022) that uses disease gene associations as groups; and miRdisNET (Jabeer et al., 2023) that uses miRNA data and miRNA target genes as groups.

This study proposes a novel method for integrating 3 types of-omics data (e.g., mRNA, miRNA, and methylation); and the corresponding tool is named as 3Mint (3 Multi-omics integrative tool). Our main objective is to utilize the statistical relationships betweenknowledge of 3-omics data in order to create groups, to perform scoring and finally to construct a classification model based on the top ranked groups. The genes within the informative groups provide insight into understanding the joint interaction of targeted genes with prominent CpGs and miRNAs. Identification of new predictive biomarkers can help the researchers to improve the prognostic and predictive accuracies.

## 2 Materials and methods

### 2.1 Datasets and preprocessing

The Cancer Genome Atlas (TCGA) (Tomczak et al., 2015)-Breast Invasive Carcinoma (TCGA-BRCA) dataset is available on the Genomic Data Commons, hosted by the National Cancer Institute (NCI). Here we focus on the microRNA (miRNA), gene expression (mRNA) and methylation datasets where the reads were mapped to GRCh38, downloaded from Xena Public Data Hubs (https://xenabrowser.net) (Goldman et al., 2020).

The BRCA molecular intrinsic subtype classes were provided by the PAM50 assay (Prediction Analysis of Microarray 50), which is originally based on 50 gene signatures (Parker et al., 2009). In this study, downloaded tumor samples are filtered for the molecular subtypes (Luminal A, Luminal B, Her2-enriched and Basal like) and classified into the following two groups: 1) Luminal group including Luminal A and Luminal B with 248 ER+/PR + PR-samples, and ER-negative group including Her2-enriched and Basal-like with 124 ER-/PR-samples, excluding normal like. The constructed groups are used for further analyses after data preprocessing steps are completed as explained in detail below.

#### 2.1.1 Omics Dataset 1 (miRNAs)

microRNA expression values in the form of log2 (RPM+1) were downloaded from the UCSC Xena browser and converted to read per million (RPM) values. 1882 miRNAs were used for further analysis.

#### 2.1.2 Omics Dataset 2 (mRNAs)

TCGA gene expression raw counts were downloaded from UCSC Xena browser and normalized with edgeR (TMM) (Robinson et al., 2010). We included 21,839 mRNAs in our analysis.

#### 2.1.3 Omics Dataset 3 (CpGs)

For methylation data level 3 beta values of 485.578 probes were obtained by Illumina Human Methylation 450 array. Probes with more than 20% missing values were removed. Then probes with an interquartile range (IQR) > 0.1 were selected. Probes targeting X and Y chromosomes (to prevent gender bias), single base extension sites (SNPs) and cross-reactive probes (Chen et al., 2013) were discarded. Selected 15,571 probes were included in our multi-omics data analysis.

### 2.2 Statistical analyses

Knime platform (Berthold et al., 2009). Variances between two groups were compared using *t*-test. Significant CpGs, miRNAs and mRNAs were filtered for *p* < 0.05; and used for further analysis. Correlation of-omics datasets were tested *via* calculating Pearson correlation in R (R Core Team, 2021). Statistically significant associations within the correlation coefficient threshold interval of (−0.6, 0.6) and with the significance level *p*-value <0.05, were selected.

### 2.3 Proposed method (3Mint)

**FIGURE 1:**
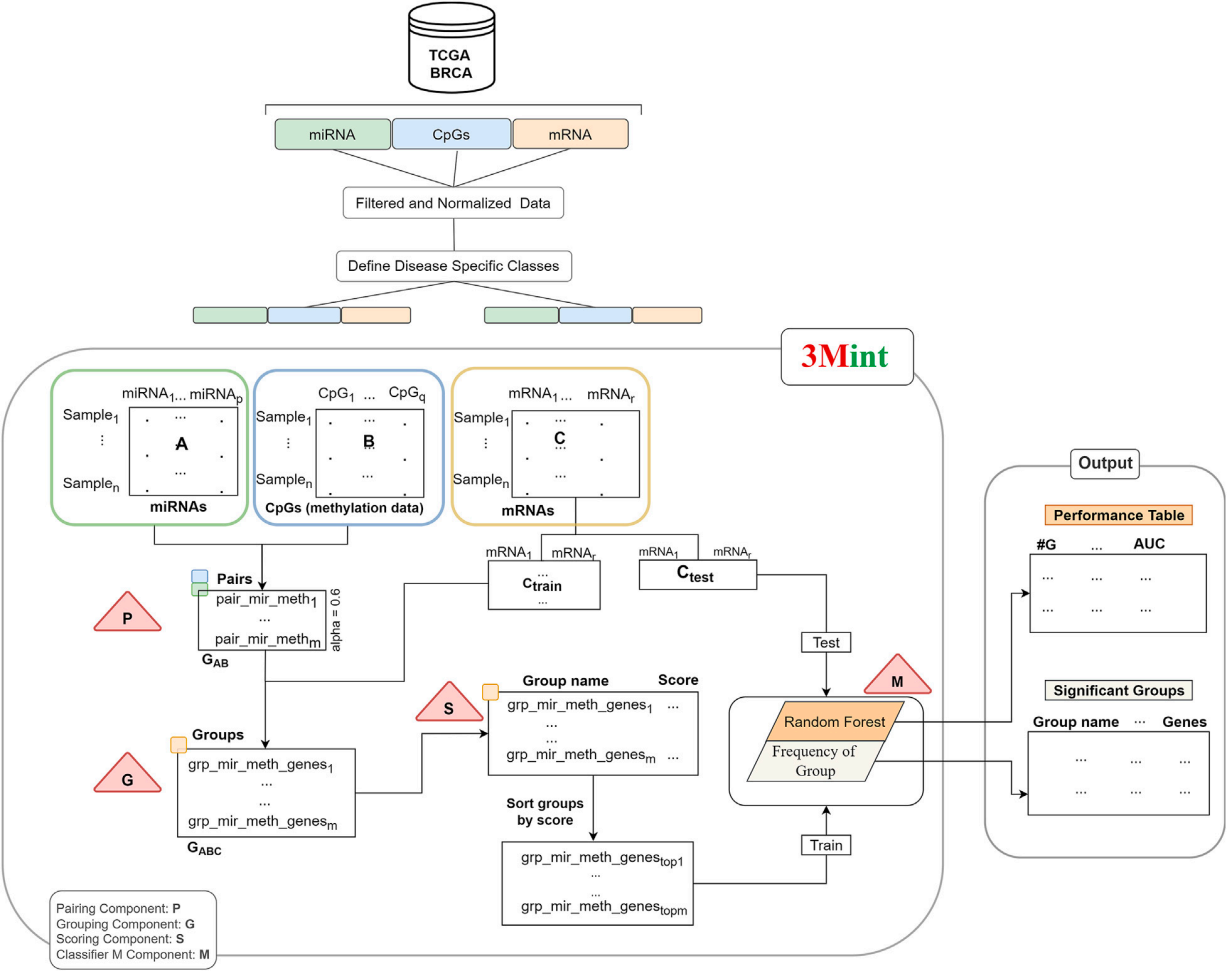
The workflow of 3Mint. The workflow consists of four main components (indicated by triangles), P) detects pairs, G) identifies groups, S) scores groups, M) creates the classifier model. The output layer consists of the 1) Performance Table, and 2) the Significant Groups Table.

The developed method using 3-omics data is explained in detail in this section. The input data required for this method is labeled data including samples from two classes. In this study, we examined samples in the Luminal group vs. samples in the ER-negative group, as defined in subsection 2.1). Our objective is to perform grouping of the features and scoring of the groups using biological knowledge. The main flowchart of 3Mint is illustrated in Figure 1.

Our method consists of four main components (P, G, S and M); and they are indicated by the triangles in Figure 1. P and G components detect sets of pairs and groups of genes, respectively. S component allows to score the groups, and M component creates the model by training the classifier.

Normalized data matrices (miRNA expression profiles and level of CpGs in methylation) are inputs of the first component. P component is applied to these datasets; and pairs of correlated miRNA-CpG features are given as output. Second component (G) creates a set of groups by using the output of the P component. A group includes highly correlated mRNAs (target data) with both miRNA and CpG pair. Group names denote the union of miRNA-CpG names, including target gene list. The third component (S) takes highly associated group sets as input and returns the scores of each group. This operation utilizes Random Forest (RF) classifier to calculate a score for each group. The groups are scored based on their ability of distinguishing samples belonging to two different classes. Subdatasets of target data that are generated *via* cross validation in each group are used as input features for the scoring component. Then, the best scoring groups are introduced into the M component and the model is created by training the RF classifier. In our experiments, we have utilized BRCA molecular subtypes as our phenotypes, denoting 2 class labels, However, it is worth noting that 3Mint allows the use of any other 2-class phenotypes.

### 2.4 Notation

Let n be the number of samples in each-omics data. Let A, B, and C denote the matrices of miRNA, CpGs and gene expression datasets, respectively. While rows represent the samples, columns are the features of these matrices. Target matrix, also named as matrix C is splitted into Ctrain and Ctest. Train matrix is used to train the classifier and fit the model following the generation of final groups with scores. Test data is used to report the performance of the model.

In component P, highly correlated miRNA-CpG pairs are defined as GAB. Let m denote the number of such pairs. In particular, let mir_methf = (ai, bj), ai in A and bj in B, where |cor (ai,bj)| > alpha. Thus GAB = {mir_methf, f = 1,m}. The correlation threshold denoted as alpha is determined by the user. The recommended default value of alpha is 0.6.

In Component G, all highly correlated mRNA features (ck) with both miRNAs and CpGs are selected for each GAB pair. Intersection of pairwise correlation of gene expression and miRNA and gene expression and CpGs with a beta threshold creates mir_meth_genes groups (GABC). In particular, define the groups mir_meth_genesf = {ck |cor (ai, ck) > beta and cor (ck,bj) > beta, ai and bj are members of the pair mir_methf, for f = 1,m}. GABC is the output set of mir_meth_genes groups and is used in the S component for scoring.

Each group consists of a unique miRNA-CpG name with single/multiple genes. Normalized gene information of each group is obtained by the target matrix (matrix C). In component S, cross validation procedure is followed. In this component, submatrices of the target matrix Ctrain of each group are generated and utilized to calculate GABC scores. Each submatrix is defined as Ctrainsubf, where f = 1,m, that contains genes that belong to the group mir_meth_genesf. A RF classifier is trained with gene expression values in each submatrix from the training set. The submatrix in the test portion is used to evaluate the performance of the model. Mean of the accuracy scores for each group is assigned as a group score.

**FIGURE 2:**
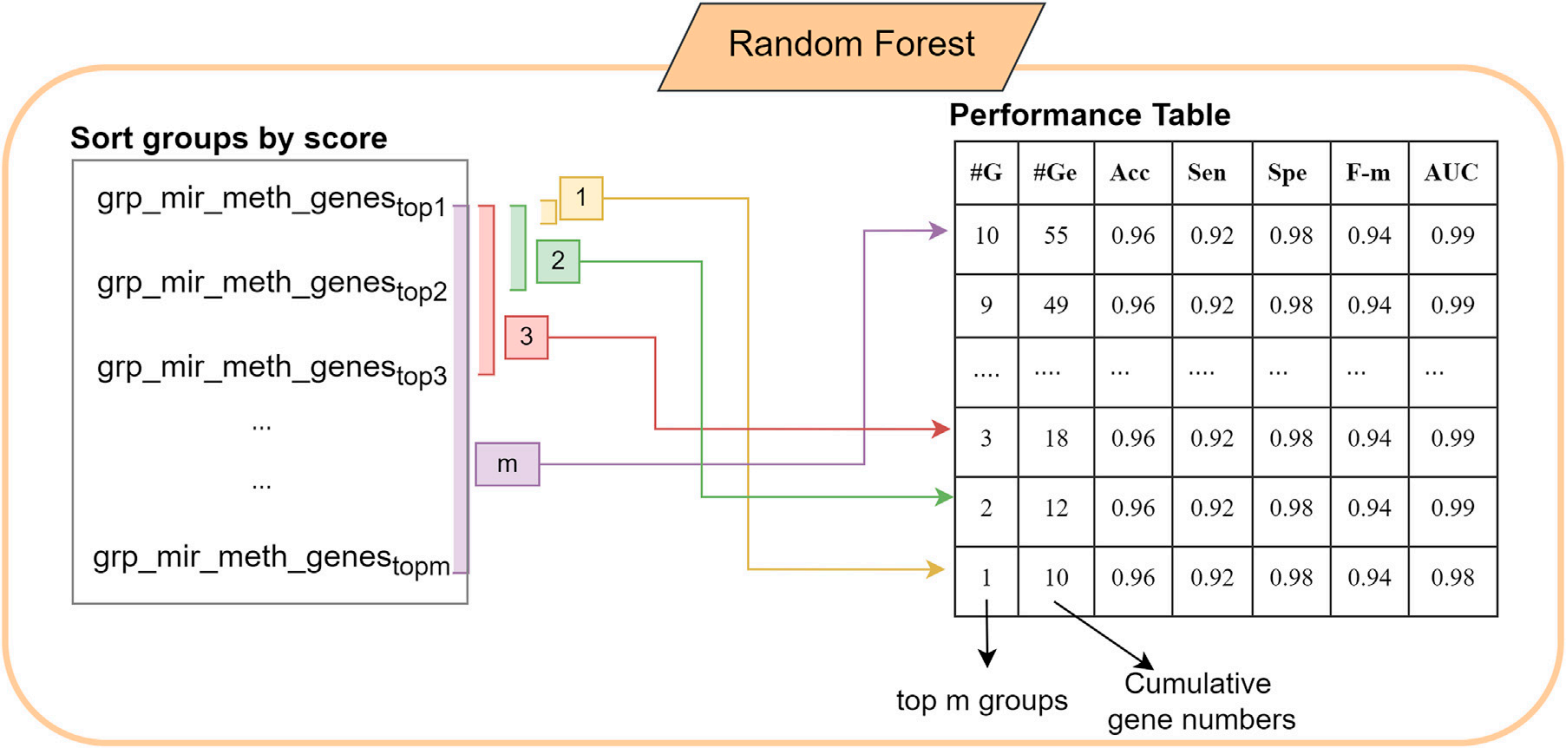
The construction of the performance table in 3Mint using the most significant groups.

The score is calculated using the chosen metrics in which the weight of the metric/metrics can be determined by the user. In this study, we use the accuracy metric as the score of each group. S function gives a list of groups and scores which can be defined as grp_scores = {(mir_meth_genesf, scoref) f = 1,m}. Following that, the list is sorted by score. In the M component a RF model is created. The first 10 best scored groups are used as the train matrix; and Ctest which is obtained by the original gene expression matrix, is used as a test matrix to measure the performance of the fitted model. M component returns performance metrics, significant groups, significant miRNAs, CpGs and genes. Performance metrics table generated by the component M includes the information on the number of cumulative groups; number of genes in each cumulative group; accuracy, sensitivity, specificity of the model. The construction of the performance metrics table is illustrated in Figure 2. Cumulative groups are created by aggregating the next high scoring group (mRNA/gene list) with previous group/groups. For example, the information presented for cumulative group #3 shows the performance metrics for the merged group including the first, second and third most significant groups, hence it shows the collective contribution of the top 3 groups to the classification procedure.

Figure 1 summarizes the workflow of the 3Mint algorithm for one split. The same process is repeated for 100 times where in each iteration gene expression data (C matrix) is splitted into train and test matrices to generate a list of mir_meth_genesf and their associated genes. The groups that are more frequently seen in 100 splits are prioritized.

## 3 Results

### 3.1 Performance evaluation of 3Mint

In this section, 3Mint classification performance results based on 100 random splits (90% train–10% test), and further statistical analyses are presented. Classification performance results of 3Mint for the BRCA molecular subtypes datasets in terms of accuracy, specificity, sensitivity, AUC (area under curve) metrics are summarized in Table 1. The symbol “±” indicates the standard error of the mean over the 100 splits. The # of unique genes denotes the number of unique genes in the cumulative groups. As shown in Table 1, the results are very strong, in particular the first group (top row) has an AUC of 0.91, accuracy of 0.88, sensitivity of 0.96, and specificity of 0.74. Most of these performance metrics improved with the accumulation of more groups (further down in the table). The best accuracy values are obtained for the cumulative group # 7. After that, the addition of more genes did not further increase any of the performance measures.

**TABLE 1:** Performance metrics of 3Mint for the BRCA molecular subtype classification problem. This output table of 3Mint also presents number of cumulative groups, number of unique genes, average gene number.

Cumulative group #	# Of unique gene	Average gene #	Accuracy	Specificity	Sensitivity	Area under curve
1	54	4.67	0.88 ± 0.05	0.74 ± 0.11	0.96 ± 0.04	0.91 ± 0.06
2	67	9.34	0.89 ± 0.05	0.75 ± 0.11	0.96 ± 0.04	0.92 ± 0.07
3	76	15.06	0.89 ± 0.05	0.77 ± 0.11	0.95 ± 0.04	0.93 ± 0.06
4	77	21.09	0.89 ± 0.05	0.78 ± 0.12	0.95 ± 0.04	0.94 ± 0.05
5	81	29.72	0.9 ± 0.04	0.79 ± 0.11	0.95 ± 0.04	0.94 ± 0.05
6	81	36.42	0.9 ± 0.04	0.8 ± 0.11	0.95 ± 0.04	0.95 ± 0.04
7	82	49.83	0.91 ± 0.04	0.82 ± 0.1	0.96 ± 0.04	0.95 ± 0.04
8	82	60.59	0.91 ± 0.03	0.83 ± 0.1	0.96 ± 0.04	0.96 ± 0.03
9	82	72.09	0.91 ± 0.03	0.83 ± 0.09	0.95 ± 0.04	0.96 ± 0.03
10	82	83.30	0.91 ± 0.03	0.83 ± 0.08	0.95 ± 0.04	0.96 ± 0.03

### 3.2 Comparative performance evaluation of 3Mint

The effect of using different classifiers in 3Mint is assessed. The performance of 3Mint using RF as the default classifier is comparatively evaluated with various classifiers such as Probabilistic Neural Network (PNN) based on the DDA (Dynamic Decay Adjustment) method, Gradient Boosted Trees (GBT) using boosting approach to build an ensemble of trees, Naive Bayes (NB) assuming that all features are independent based on Bayes’ law. The performance of the models for the classification of Luminal and ER-negative groups are evaluated over 10- fold Monte Carlo cross-validation; and the results are summarized in Supplementary Table S1. The performance of the RF, PNN, NB, and GBT models at different threshold values as a result of Area Under Curve ROC analysis are also visualized in Supplementary Figure S1. The accuracy of quantitative tests are given as AUC scores. The discrimination ability of the models for the best scored group is GBT. Random Forest and Naive Bayes follow GBT with very close AUCROC scores. All tested classifiers resulted in similar high AUC scores. The average AUC score of the RF model in our experiments is 0.97, and the RF model resulted in adequate accuracy (0.91) for discriminating positive cases (Luminal group) from negative cases (ER-negative group). In RF, the interpretation of the tree model is simple and the model can be easily transformed into a ruleset. Also, RF is one of the widely used supervised machine learning algorithms for dealing with omics data in the analysis of biological processes and/or disease progression of complex diseases (Lind and Anderson, 2019; Toth et al., 2019; Uddin et al., 2019; Quist et al., 2021). For these reasons, in this study, we focused on the results obtained using the RF classifier.

Additionally, we have compared the performance of 3Mint using three omics data (mRNA, miRNA, and methylation) with single-omics data analysis and with 2-omics data analysis. To this end, we have conducted a similar experiment using only mRNA dataset; and a similar experiment with miRcorrNet tool (Yousef et al., 2021a) using 2-omics datasets (miRNA and mRNA datasets) for the BRCA molecular subtype identification problem. For the single omics data analysis, all available gene expression information, in total 21.839 gene expression values are used to create a random forest model. The performance of the model is evaluated using 10-fold Monte Carlo cross validation (90%–10% split). For two omics data analysis, miRcorrNet tool which conducts ML–based analysis of miRNA and gene expression profiles, are utilized. Groups including target genes associated with each miRNA are generated based on correlation analysis of mRNA-miRNA expression profiles. The ranked groups are used to perform classification task and disease specific biomarkers are identified with the miRcorrNet tool.

Supplementary Table S2 presents the comparative evaluation of 3Mint with single-omics data analysis and two-omics data analysis for the classification of Luminal and ER-negative groups. In this table, while the top group indicates the best scoring groups, top 10 cumulative groups refer to the 10 best significant groups for the 3Mint and mirCorrNet analysis. Although the performance metrics of 3Mint are comparable with miRcorrNet, miRcorrNet results in higher numbers of genes for the BRCA molecular subtype identification problem. In other words, considering the top 10 groups having 13.6 and 38.2 genes on average in 3Mint and miRcorrNet respectively, one can conclude from Supplementary Table S2 that 3Mint classifies the BRCA molecular subtypes with lower number of genes when compared to miRcorrNet. This finding implies that the incorporation of methylation data in 3Mint yields a much more focused analysis. Similarly, the performance metrics of 3Mint and miRcorrNet methods are comparable with single-omics data analysis (using 21.839 mRNAs). The addition of miRNA and methylation information enables the classification of BRCA molecular subtypes with a very low number of genes even without loss of performance (in terms of AUC metric). In this study, our main aim is the investigation of new biological insights into how the molecular mechanisms (proliferation, differentiation, survival, etc.,) of the interested disease are regulated by focusing on the interaction of gene, miRNA and CpG features. The prominent features associated with the top groups are utilized in the enlightenment of the cellular signaling and molecular interactions of markers in complex diseases.

### 3.3 Clinical analysis

In order to evaluate the relationship between the predicted classes and the clinical data, we have performed survival analysis for each classification group, using the available survival data at TCGA. Survival over time for defined classes (ER-negative and Luminal) is computed with Kaplan-Meier estimate. The survival time refers to the number of days from the diagnosis to death, last contact or end of the study. The log-rank test is applied to compare the survival differences of classes. The time dependent number of patients at risk for ER-negative group and Luminal group are provided in Supplementary Figure S1. The survival rates of true and predicted classes are quite similar (*p*-value: 0.77, 0.89 respectively).

### 3.4 Most significant groups that are identified by 3Mint on BRCA molecular subtype datasets

The analysis of the BRCA molecular subtype datasets using 3Mint revealed associated biomolecular groups, differentially expressed mRNAs/genes, miRNAs, and CpGs. We have further investigated the relationships between these biomolecules. To this end, Supplementary Table S3 presents 10 most significant groups that are obtained *via* running 3Mint on the BRCA molecular subtype datasets. In this table, the groups refer to the miRNA/CpG groups; and the groups are sorted by their frequencies (number of times the group is identified as significant in the 100 splits). The groups with frequency numbers smaller than or equal to 5 are filtered out before creating the significant groups. Average score denotes the scores ranging from 0 to 1 are computed by the Scoring Component and averaged over 100 splits. The higher the score, the better ability of the group in terms of distinguishing the classes of the BRCA molecular subtypes. Average rank expresses the mean of the assigned rank of each specific group in each split. Lower rank indicates stronger statistical significance. List of target genes of each group and the number of these genes in the union set are given under the columns Associated genes and # Associated genes, respectively. Furthermore, in each split, a gene list is generated by collecting the union of the genes targeted by the group. In Supplementary Table S3, these gene numbers in the list for each group are summarized as the minimum and maximum gene numbers (Min and Max Gene #) associated with the group. Median Gene # is calculated by computing the median of the number of genes in those gene lists. One can observe from Supplementary Table S3 that the most frequently detected group, hsa-miR-20a_cg02370232, was reported in 90 splits, and this group has 16 associated genes. Functional enrichment analysis is performed to derive over-represented GO categories of these identified genes using DAVID (Sherman et al., 2022). Supplementary Table S4 presents the functional enrichment of 16 genes in the most frequently detected group (hsa-mir-20a_cg02370232). In the GO MF (molecular function) category, transcriptional regulation activity of BCL11A (B-cell lymphoma/leukemia 11A) with FOXC1, OTX1 and ZNF232 is detected. Cellular division activity of the genes RNF8, PIMREG, and AURKB are identified with the GO:0051301 (cell division) term. The functional enrichment analysis of the genes within the top 5 appeared groups are also shown in the groupGO_analysis Supplementary Materials.

### 3.5 Most significant miRNAs that are revealed by 3Mint on BRCA molecular subtype datasets

Supplementary Table S5 presents the top ten most significant miRNAs that are identified by 3Mint on BRCA molecular subtype datasets, including related summaries and lists of associated CpGs and genes. The frequency shows the number of splits (out of 100) where that miRNA appears in at least one group (where the group consists of a miRNA-methylation pair). The total frequency is the total number of groups in which the miRNA appears in the group. Additionally, miRNA associated genes and CpGs are listed in Supplementary Table S5. The full lists of splits in which this miRNA appears, and the corresponding group ranks are presented in the last two columns.

Supplementary Table S5 suggests that three miRNAs (hsa-miR-20a, hsa-miR-17, and hsa-miR-19a) involve heavily in the BRCA molecular subtype identification problem. These miRNAs appear in more than half of the splits, and in multiple groups within each split. They also have relatively large sets of associated genes and CpGs indicating a strong regulatory network, which may be a distinguishing feature for the BRCA molecular subtype. The miRNAs appearing in fewer groups tended to appear less frequently, indicating the occasional appearance of that miRNA for BRCA (consistent with the cancer’s very heterogeneous nature). Two major exceptions are hsa-miR-18a and hsa-miR-135b, which appear in few splits, but in many different groups (i.e., they are associated with several CpGs) in those few splits. Interesting future work is an investigation into potential biological drivers of this phenomenon. A good starting point will be the latter miRNA, focusing on those specific splits. Also of interest is the patterns of joint appearance of these miRNAs.

### 3.6 Most Significant CpGs that are detected by 3Mint on BRCA molecular subtype datasets

Supplementary Table S6 presents the top ten most significant CpGs that are identified by 3Mint on BRCA molecular subtype datasets, including related summaries and lists of associated miRNAs, mRNAs. This table is prepared in a similar manner with the miRNA table shown in Supplementary Table S5. Contrary to the occurrence of significant miRNAs in several groups in each split, significant CpGs appeared in at most 2 groups in each split. Supplementary Tables S5, S6 imply that while six significant miRNAs contribute to the regulatory mechanisms of BRCA molecular subtype characterization, more than 10 significant CpGs have a potential role in this mechanism. In this context, the dominance and high potency of miRNAs over CpGs can be inferred from this table. One can observe from Supplementary Table S6 that the two most significant CpGs (cg02370232, and cg06282596) share similarities in terms of their occurrence, average scores, average ranks, number of associated genes and number of associated miRNAs (19 common genes out of 23–24 genes, and 5 common miRNAs out of 6 miRNAs). These results point the way towards a potential further investigation of similarly behaved CpGs on their cellular functionality, targeting sites and potential complementaries. It is worth noting that most of the CpGs involve the miRNAs hsa-miR-20a and hsa-miR-17. An important exception is cg24296761, which is found to be associated with hsa-miR-18a and hsa-miR-106b, not hsa-miR-20a and hsa-miR-17. This CpG occurs in only 10 splits, but it has a strong effect when it appears as indicated by the very low average rank.

### 3.7 Most significant mRNAs/genes that are highlighted by 3Mint on BRCA molecular subtype datasets

Table 2 gives a gene centric view of the results, showing the distribution of the identified genes over the groups. This table gives the 10 most significant genes with relevant summary statistics. The most prevalent genes across the significant groups were BCL11A, SRSF12, RASAL1, and L3MBTL4. We investigated the biological relevance of these genes in the literature. For instance, BCL11A (B-cell lymphoma/leukemia 11A) gene, which encodes a regulatory C2H2 type zinc-finger protein, is commonly characterized as a transcriptional repressor. It was reported that BCL11A has a potential role in tumor proliferation and metastasis through the Wnt/β-catenin signaling pathway in BRCA (Zhu et al., 2019). It was noted that BCL11A can be used in triple negative BRCA (TNBC) therapy as a potential biomarker, but the precise mechanism of the gene on TNBC is not clear yet (Wang et al., 2020). Just a handful of studies have focused on SRSF12 (serine and arginine rich splicing factor 12) gene, which is a member of the SR (Serine–arginine)-rich splicing factors in BRCA. A study revealed that the upregulation of SRSF12 and other family members SRSF3 and SRSF2 were detected in 20%–40% of TNBCs (Park et al., 2019). Furthermore, a protein encoded by the RASAL1 (RAS protein activator like 1) gene suppresses RAS protein function; and thereby serves as a negative modulator of the RAS signaling pathway. It was demonstrated that RASAL1 regulates hypoxia inducible factor-1α through the Akt/Erk pathways (Huang et al., 2017). In our analysis, L3MBTL4 (histone methyl-lysine binding protein 4) gene, which is known as a tumor suppressor gene, is identified as another potentially important gene for the classification of Luminal and ER-negative groups. A study reported the reduced L3MBTL4 gene expression in non-basal breast tumors (Addou-Klouche et al., 2010).

**TABLE 2:** Top ten most significant mRNAs/genes that are detected using 3Mint on BRCA molecular subtype dataset.

Genes	Freq of genes	Total freq in each group	Average score	Average rank	Group list	Group #	Rank list	Split list
BCL11A	95	752	0.89	7.00	hsa-miR-20a_cg24051242, …	70	1, 2, 3, 4, 1, 2, 3, 3, 4, 4, 5,…	0, 0, 0, 0, 1, 1, 1, 1, …
SRSF12	77	483	0.90	6.10	hsa-miR-17_cg12427162, hsa-miR-17_cg02370232, …	65	1, 2, 3, 3, 4, 4, 1, 2, 3, 3, 4, 5, …	1, 1, 1, 1, 1, 1, 4, 4…
L3MBTL4	49	171	0.89	8.12	hsa-miR-20a_cg12427162, …	38	1, 2, 3, 4, 5, 6, 6, 9, 2, 4, 4,…	4, 4, 4, 4, 4, 4, 5, 5,…
RASAL1	42	111	0.90	5.06	hsa-miR-19a_cg24051242, hsa-miR-19a_cg02370232, …	21	1, 2, 3, 4, 1, 1, 2, 2, 13, 1, 1, 2, 1, …	5, 5, 5, 5, 6, 7, 7, 7, 7, 9,…
RNF8	42	126	0.89	5.85	hsa-miR-20a_cg24051242, hsa-miR-17_cg12427162, …	20	1, 1, 1, 4, 4, 6, 3, 9, 2, 4, 5, 5, 8…	0, 1, 4, 4, 6, 6, 7, 7, 10, …
APBA2	37	93	0.89	6.38	hsa-miR-19a_cg24051242, hsa-miR-17_cg24051242, …	15	1, 9, 1, 3, 13, 6, 1, 2, 4, 5, …	7, 7, 9, 9, 12, 13, 16, 22, 24,…
CDCA7	31	75	0.91	4.33	hsa-miR-17_cg26242687, hsa-miR-20a_cg12427162, … …	23	3, 1, 3, 4, 1, 1, 9, 2, 4, 1, 4, 8 …	1, 4, 4, 4, 5, 7, 7, 10, 10, 12, 12, 12, …
RPIA	29	84	0.91	7.14	hsa-miR-19a_cg24051242, hsa-miR-19a_cg24051242, …	24	1, 1, 3, 9, 2, 5, 1, 2, 3, 4, 5, 6, 7, 8, …	5, 7, 7, 7, 10, 10, 12, 12, 12,…
CDKN2A	27	82	0.91	6.40	hsa-miR-20a_cg24051242, hsa-miR-20a_cg24051242, …	24	1, 3, 2, 5, 1, 2, 3, 4, 7, 8, 10, 11, 12, …	0, 7, 10, 10, 12, 12, 12, 12, 12, 12,…
PIMREG	24	114	0.90	6.93	hsa-miR-19a_cg24051242, …	26	1, 2, 2, 3, 4, 5, 6, 7, 8, 9, 10, 11, …	7, 7, 7, 7, 7, 7, 7, 7, 7, 7, 7, 7, 7, 10, 10, …

### 3.8 Patterns of co-occurrences for the identified groups, miRNAs, CpGs, and genes

**FIGURE 3:**
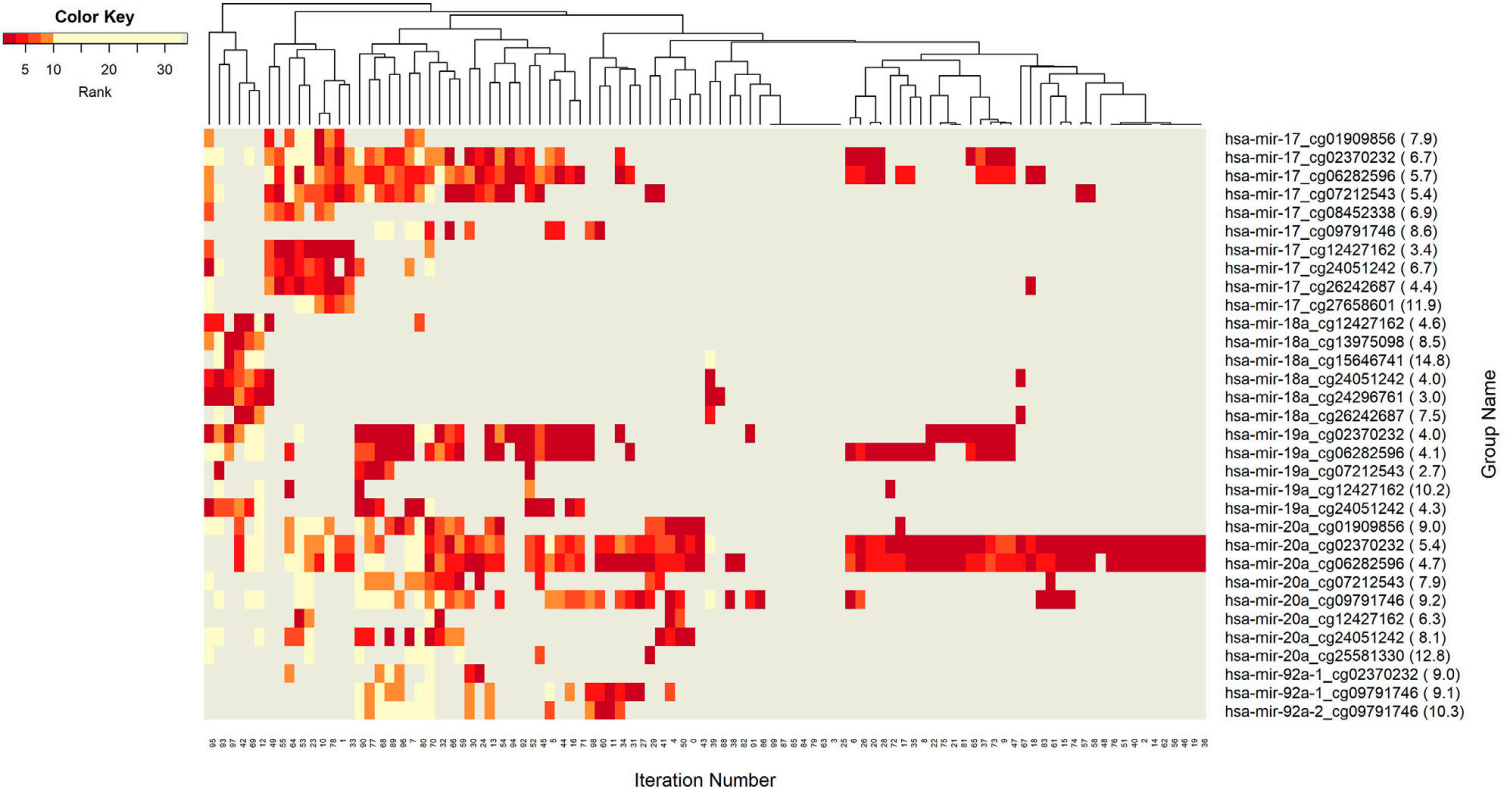
Heatmap of Group features with rank information for each split. The heatmap reveals patterns of co-occurrence of groups over different splits. Color key encodes the rank of each group, where 1 indicates the top rank. Gray indicates non-detection of that group for the corresponding split.

**FIGURE 4:**
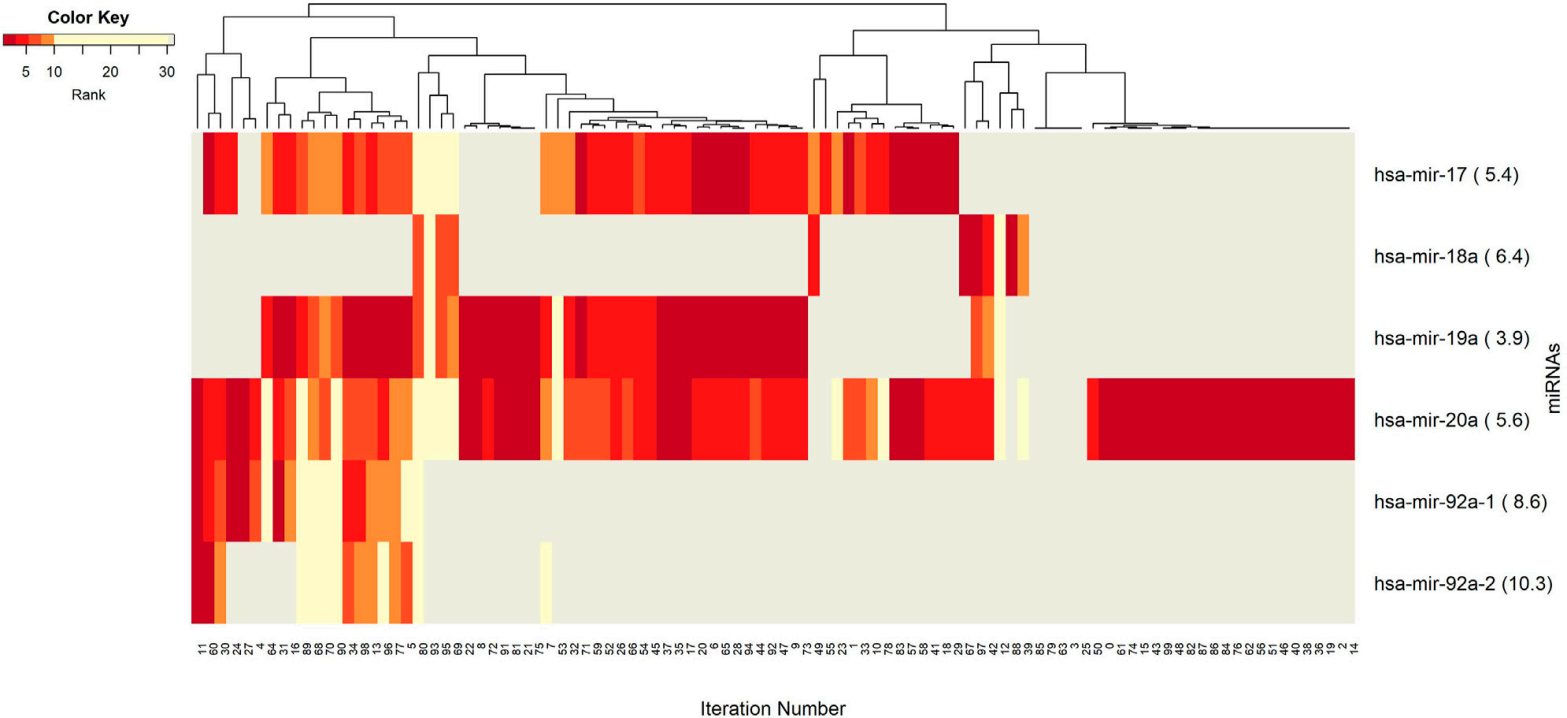
Heatmap of miRNA features with rank information for each split. Co-occurrences over the splits of the group component miRNAs are revealed. The color key encodes the rank of each miRNA, where dark red indicates the top ranking. Gray displays non-detected miRNAs for the corresponding split.

**FIGURE 5:**
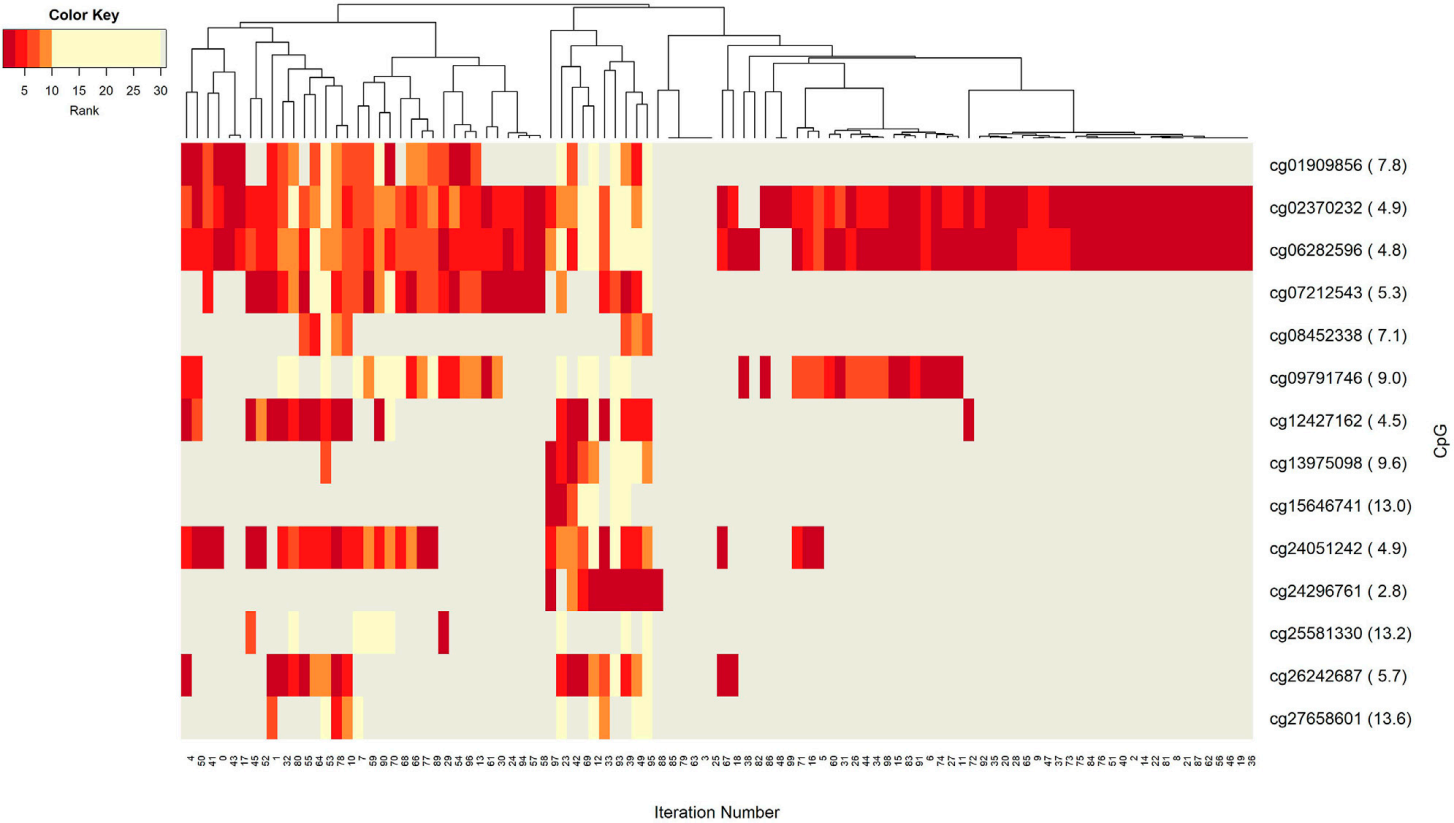
Heatmap of CpG features with rank information for each split. Heatmap illustrates co-occurrence patterns of the group component CpGs over splits. Color key encodes the ranking of each CpG with a spectrum from dark red to yellow. Gray displays non-detected CpGs for the corresponding split.

**FIGURE 6:**
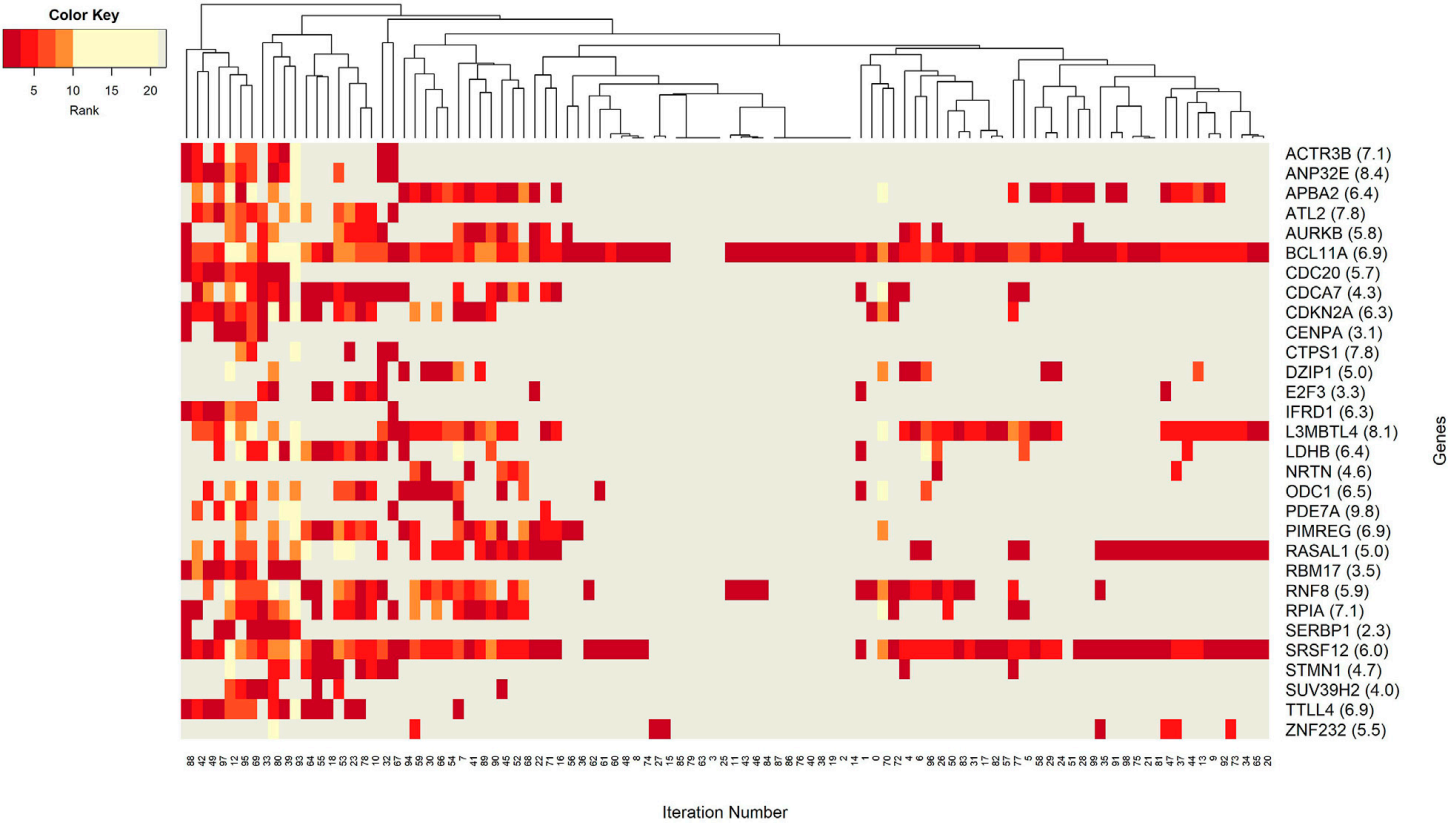
Heatmap of gene features with rank information for each split. Heatmap reveals patterns of co-occurrence of genes over different splits. Rank of each gene is illustrated using colors. Gray color is used to denote non-detected groups.

The networks of significant-omics markers detected *via* concurrent analyses can provide a robust view of the dysregulated mechanisms, explaining the molecular subtypes of a disease. A collective interconnection of the features based on the developed 3Mint tool can provide potential relationships among the hidden layers of the-omics data. Along this line, we analyzed the patterns over the 100 splits for the detected groups, miRNAs, CpGs and genes. In Figures 3–6, we have visualized these patterns as heatmaps where splits are shown in the columns, and feature names (group, miRNA, CpG or gene) are shown in the rows. In order to place splits with similar patterns next to each other, the columns (i.e., splits) are hierarchically clustered using the rank information, calculating the Euclidean distance, and applying average linkage. Additional insights about the relation between columns come from the dendrogram. The color key denotes the ranking of features in a scale from most highly ranked (shown in dark red) to the least (shown in yellow). Gray areas indicate non-detected features for the corresponding split. The numbers in parentheses following the feature name shows the average rank of the group over 100 splits.

Figure 3 demonstrates the significant players (groups) with dark color, indicating that these groups are most frequently seen over 100 splits. In this analysis, the groups with 5 or fewer appearances are omitted. The groups hsa-miR-20a_cg02370232, and hsa-miR-20a_cg06282596 are found as the two most important players (Supplementary Table S3). These groups have a very similar pattern in terms of splits (Figure 3) indicating a strong co-occurrence network. Note that these two CpGs also showed up in the four following groups, i.e., hsa-miR-17_cg02370232, hsa-miR-17_cg06282596, hsa-miR-19a_cg02370232, and hsa-miR-19a_cg06282596, which appear together with the dominant groups on the left side of the heat map, but much less frequently on the right side, indicating occasional co-occurrence. Two small clusters on the left of the heatmap suggest small sets of groups containing hsa-miR-17 and hsa-miR-18a, which only appear in a few splits, suggesting a fairly rare but also important phenomenon which may be worth deeper investigation. The small cluster of columns near the middle indicates that a few of the splits resulted in the detection of no groups.

Next we aim to create a more detailed view, explaining the role of each particular component (miRNAs, CpGs, and genes) from the 3-omics dataset, by generating similar heatmaps, derived from Supplementary Table S3; Figure 3.

The co-occurrence patterns of the miRNAs, which appear in the groups in Figure 3, are illustrated in Figure 4. In other words, deeper insights into the patterns of miRNA appearance over the 100 splits comes from the heatmap visualization (Figure 4). Color encodes the average ranks of the groups where the groups include that particular miRNA. The most frequently appearing miRNA, hsa-miR-20a, with an average rank of 5.6 seems to be a hub miRNA. Other potentially important players include hsa-miR-19a, hsa-miR-17 which have high ranks positioned in similar splits. The overlapped splitting patterns of these two miRNAs suggest a possible cooperation among these two miRNAs. The least frequently seen miRNAs, above the frequency threshold, hsa-miR-92a-1 and hsa-miR-92a-2 have very similar split patterns positioned in the bottom left of the table which is not surprising as they are isoforms. These patterns again suggest the presence of relatively small clusters in the data, which requires deeper investigation.

Insight into the patterns of occurrence of CpGs over splits come from the heatmap view. Figure 5 represents the colored patterns of significant CpGs, where the color is assigned based on the split numbers. Strong overlapped patterns of cg02370232 and cg06282596 suggest similar functional mechanisms among these two CpGs. As mentioned in the significant groups table, these CpG has strong interaction with hsa-miR-20a. The co-occurrence of cg12427162 and cg24051242 is not frequent enough, but we can still argue about their contribution to BRCA molecular subtype classification.

Figure 6 focuses on the identified genes, which could have a potential role in distinction of BRCA molecular subtypes. More specifically, the co-occurrence patterns of the genes in different splits is visualized in the heatmap. One can imply that the BCL11A gene may have a key role in training of the model because it appeared in 95 out of 100 splits. High average score and high average rank enhances the idea that BCL11A is the most prominent gene for the classification of BRCA molecular subtypes. Similarly, the gene SRSF12 appears in every split that includes BCL11A. The split patterns in the heatmap show that both BCL11A and SRSF12 are prevalent in 100 splits. A deeper investigation reveals that these two most frequently occurring genes, BCL11A and SRSF12, both appear in 59 groups (out of 70 groups with BCL11A and 65 containing SRSF12). This observation suggests that these two genes may have same/similar targeting sites. Additionally, with an average rank of 6.9 and 6.0, respectively, these genes could have substantial effect on the construction of significant groups. Significant co-occurrence relationships of the genes increase their dominance in classification. A relatively less frequently seen gene ZNF232 with an average rank of 5.5, also influenced the classification performance. Moreover, one can observe from Figure 6 that APBA2, RASAL1, and L3MBTL4 have similar patterns in the heatmap. Hence, to better understand BRCA molecular subtypes, the functional relationships among these genes may be worth further investigation.

### 3.9 Analysis of the groups based on their shared genes

**FIGURE 7:**
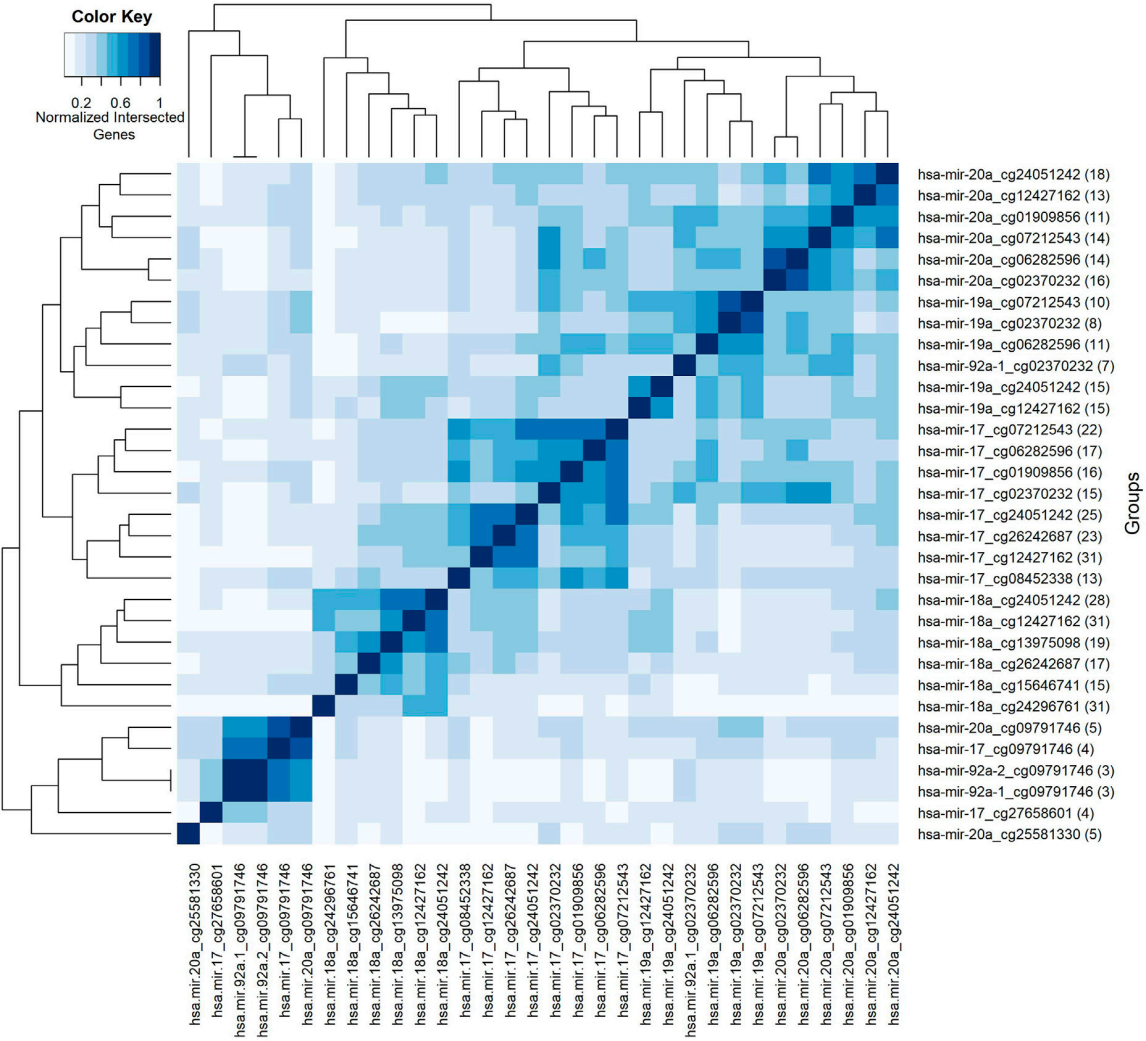
Heatmap of group features based on normalized intersected genes among each pair of groups. Color key encodes normalized intersected gene numbers for each pair of groups. Scale 1 indicates group pairs with most normalized shared gene numbers.

So far, we have presented our findings about significant features, depending on their involvement in different splits within the classification experiment. We have performed additional analyses to understand the collective behavior of the groups based on their shared genes. For this analysis, shared genes are utilized to construct a group-group similarity matrix. The number of genes in the intersection of each pair of groups is normalized by the total number (i.e., number in the union). Groups are hierarchically clustered based on Euclidean distance and based on average linkage, to highlight relationships between them. Such relationships go beyond mere pairwise analysis, by revealing clusters of groups. To this end, the heatmap in Figure 7 visualizes the collective behavior of the groups based on their shared gene information. The scale of the color reflects normalized intersecting gene counts within the range of (0–1). The numbers shown in parentheses following the group names show the gene number in the group. The group names are shown in both *x* and *y*-axes. Five blocks with different sizes are visually apparent along the main diagonal in the heatmap, with two distinct groups appearing at the bottom. Calling each of the latter two as a “block”, leaves us with seven total blocks, whose common gene sets are presented in Table 3.

**TABLE 3:** Shared gene lists in different blocks.

Block number	Shared gene list
1	AURKB, BCL11A, FOXC1, L3MBTL4, PIMREG, RASAL1, RNF8, SRSF12
2	BCL11A, L3MBTL4, PIMREG, RASAL1
3	ATL2, AURKB, BCL11A, L3MBTL4, PIMREG, SOX9-AS1, SRSF12
4	ACTR3B, ANP32E, ATL2, BCL11A, IFRD1, FAM171A1, SRSF12
5	BCL11A, LDHB, L3MBTL4
6 (hsa-miR-17_cg27658601)	BCL11A, ATL2, TTLL4, L3MBTL4
7 (hsa-miR-20a_cg25581330)	BCL11A, CDKN2A, NRTN, SRSF12, ZNF232

The first block shows that the groups with the miRNA hsa-miR-20a contain many genes in common. The same trend holds for the second, third and fourth blocks for the miRNAs named hsa-miR-19a, hsa-miR-17, and hsa-miR-18a, respectively. Whereas the fifth block reveals a common gene set shared by groups containing the methylation probe cg09791746. Note that these groups have much smaller gene sets, suggesting that the size of the gene list may also contribute to the clustering of the groups. The intersection of the gene sets for these four groups are BCL11A, L3MBTL4, and LDHB. The gene L3MBTL4 has a role in different Gene Ontology (GO) biological process (BP) terms including chromatin organization and regulation of gene expression. BCL11A is involved in regulation of transcription by RNA polymerase II. The other shared gene LDHB in the fifth block is implicated in pyruvate and lactate metabolic processes, as defined by GO BP. The singleton blocks at the bottom have somewhat distinct gene sets compared to the other blocks. In particular the group hsa-miR-20a_25581330 is associated with BCL11A, SRSF12, NRTN, CDKN2A, and ZNF232 genes; and hsa-miR-17_cg27658601 is associated with BCL11A, ATL2, TTLL4, and L3MBTL4 genes. As seen in Table 3, there are several genes in the lists for these singleton blocks which do not appear in any of the shared gene lists for the first 5 blocks. One is TTLL4 which is only detected in group hsa-miR-17_cg27658601, and is annotated in microtubule cytoskeleton organization and protein modifications. Another unique gene NRTN is included in MAPK (Mitogen-Activated Protein Kinase) cascade activity. The gene ZNF232 is annotated in gene expression by RNA polymerase II. Finally CDKN2A is involved in protein polyubiquitination and rRNA processing.

### 3.10 Most frequently observed genes within most frequently seen groups

**FIGURE 8:**
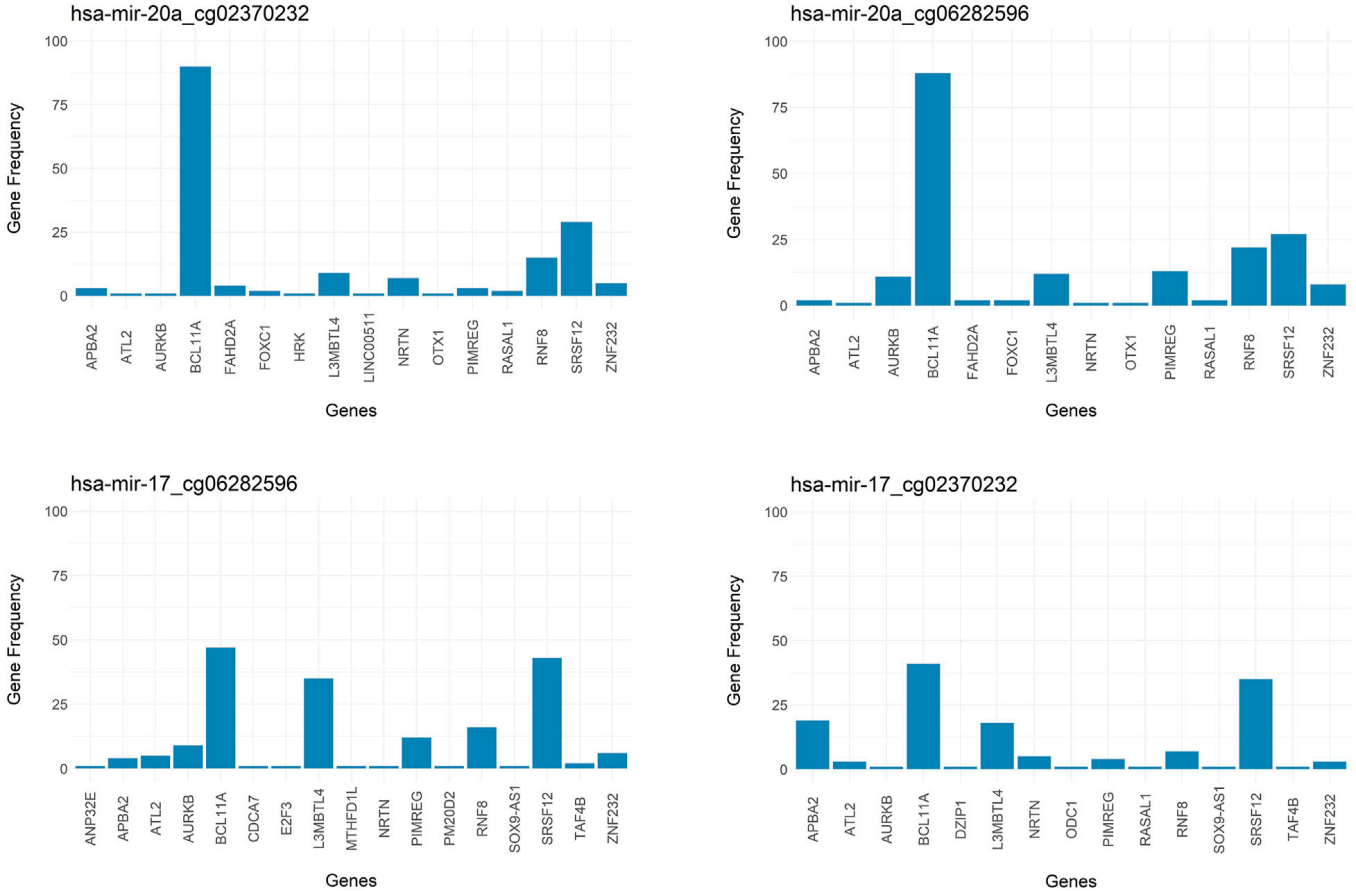
Bar graph of frequency of genes over splits for most significant groups. Targeted genes by the groups of **(A)** hsa-miR-20a_cg02370232, **(B)** hsa-miR-20a_cg06282596, **(C)** hsa-miR-17_cg06282596, **(D)** hsa-miR-17_cg02370232 are represented in frequency level.

Additional insight into the output of 3Mint comes from studying gene frequencies. Figure 8 presents gene frequencies as bar charts for the four groups most frequently seen over the 100 splits. Bars correspond to genes, and the bar height represents the number of times this gene appeared in a split for this group. BCL11A and SRSF12 are the most frequently observed members of these four groups, where BCL11A appears much more frequently in the first two groups. The first two groups have very similar gene sets and frequency patterns, suggesting a common biological role of the CpGs cg02370232 and cg06282596 with respect to hsa-miR-20a. These methylation probes target differentially methylated CpG sites located in the gene SORBS1. To understand the relationships between the genes appearing in the top 2 panels of Figure 8, we check the affected GO terms for the union of these genes, together with SORBS1 using WebGestalt (Liao et al., 2019). This analysis resulted in sets of genes with similar ontologies. The most frequently observed gene set was ATL2, BCL11A, HRK, SRSF12, AURKB, and SORBS1. This gene set has a role in cellular component biogenesis, as defined in the GO BP category. The next most numerous gene set was FOXC1, RASAL1, OTX1, BCL11A, and NRTN; which are annotated with the following GO terms: Anatomical structure morphogenesis, growth, regulation of signaling. Hence, 3Mint seems to have established new connections between these gene sets and hsa-miR-20a. Furthermore, when we have checked for the possible associations of these genes with hsa-miR-20a in literature, we found that (Huang et al., 2019b) experimentally validated the relation of ATL2, RNF8, ZNF232, and SRSF12 genes with hsa-miR-20a.

As mentioned above, and as shown in Figure 8, the most frequently observed CpGs are cg02370232 and cg06282596, which target the same SORBS1 gene in chromosome 10. This finding explains their strong overlapped patterns in Figure 5. SORBS1 acts as a potential suppressor in tumorigenesis and low levels of the gene enhances the invasive ability of BRCA cells through activation of c-Jun N-terminal kinase (JNK) (Song et al., 2017).

Note that the groups in the bottom two panels of Figure 8 are associated with hsa-miR-17 and with the same two CpGs (cg02370232 and cg06282596). Furthermore, the gene sets (especially those with higher frequencies) are very similar. Hence, the above line of reasoning suggests that hsa-miR-17 is also connected to the above mentioned two gene sets.

These results are also consistent with those of (Concepcion et al., 2012). They reported that the MicroRNA-17-92 Family, also named as “miR-17/92 cluster”, is located in the locus of the non-protein-coding gene MIR17HG, which is dysregulated in cancer tissue, thereby affecting cell cycle, apoptosis and other crucial processes. Note that all of the miRNAs appearing in Figure 4 (hsa-miR-17, hsa-miR-18, hsa-miR-19, hsa-miR-20, and hsa-miR-92) are members of this cluster. A study conducted by Moi et al., 2019 has revealed that the miR-17/92 cluster is overexpressed in aggressive breast tumors; and pointed out pivotal regulatory functions of miR-17-92 cluster and the miR-17 family in malignancy in BRCA. This is consistent with our findings, implying the molecular differences between the BRCA Luminal group (Luminal A and B) with ER-negative group (Her2 enriched and Basal).

### 3.11 Contribution of Significant CpGs and their target genes to breast cancer molecular mechanisms

The genes which map to the significant CpG sites are used to understand the contribution of the methylation information to breast cancer tumorigenesis and progression mechanisms. To this end, the frequently identified CpGs in our method and their target genes are investigated. Supplementary Table S7 shows the significant CpGs, the target genes which map to these CpG sites, and the genomic coordinates of these CpGs. Here a significant CpG site refers to a CpG which is identified in a group that is detected at least five times over 100 iterations in our analysis. The associations of these identified target genes with breast cancer are reported in literature as follows. SORBS1 acts as a potential suppressor in tumorigenesis and low levels of the gene enhances the invasive ability of BRCA cells through activation of c-Jun N-terminal kinase (JNK) (Song et al., 2017). ADAMTSL5 loci is targeted by estrogen receptor alpha (ERα) (Zhang et al., 2021), while MCF2L Antisense RNA 1 promotes the transcriptional activity of yes-associated protein (YAP) to enhance the proliferation and metastasis in Breast cancer (She et al., 2022). LRP5 activity associated with the β-catenin signaling pathway in breast cancer is reported in the literature (Björklund et al., 2009). Similarly, NOTCH1 regulates cell cycle and accelerates triple-negative breast cancer (TNCB) (Miao et al., 2020). The long non-coding RNA FOXP4-AS1 promotes cancer cell proliferation in different types of human cancers (Wu et al., 2019; Zhao et al., 2020). Furthermore, the tumor suppressor gene ZNF750 (Cassandri et al., 2020) and the oncogene WWTR1 (Cordenonsi et al., 2011) regulates cell migration and invasion.

### 3.12 Regulatory network for BRCA molecular subtype identification

To understand the differential regulatory mechanisms between Luminal and ER-negative groups, the 10 most frequently appeared groups over 100 iterations are further analyzed. The genes in these groups, the target genes which map to the CpG sites that are identified in top 10 groups, and miRNA target genes are used to construct an association network as illustrated in Supplementary Figure S3A, using STRING (Szklarczyk et al., 2021). The network represents the proteins as nodes and associations between the proteins as the links. The network is enhanced with colors encoding functionally enriched pathways (listed in Supplementary Figure S3B). The functional annotation details of the query proteins are given in Supplementary Table S8. As shown in Supplementary Figure S3B, the p53 signaling pathway, Cell cycle, MicroRNAs in cancer, and Breast cancer pathways are identified within the top 10 most significant KEGG pathways. Multi colored nodes indicate that the protein is mapped to several pathways. For example, the CDKN2A gene, which is identified by 3Mint, appears in eight different pathways among the top 10 important KEGG terms. This hub gene plays an important role in the cell cycle and the variants cause dramatic changes in the protein function, which leads to significant association of the variants with breast cancer (Aftab et al., 2019). Another prominent protein NOTCH1 is identified in our analysis and it functions as a regulating factor in proliferation and apoptosis. Hypomethylation of the gene causes over-expression of the NOTCH1 protein in Notch signaling pathway (Sun et al., 2016). ANP32E, AURKB, BCL11A, CDK2AP1, LDHB, and RNF8 are other genes that are identified in the 10 mostly appeared groups and visualized in the network in Supplementary Figure S3A. While ANP32E, AURKB and BCL11A induce tumor progression in triple-negative breast cancer cells (Khaled et al., 2015; Xiong et al., 2018; Huang et al., 2019a), downregulation of CDK2-AP1 enhances tumor growth through cell cycle regulation (He et al., 2014). It was reported that RNF8 promotes breast cancer metastasis *via* enhancing Epithelial-mesenchymal transition (EMT) (Kuang et al., 2016). In another study, LDHB was found to be linked to breast cancer by controlling early tumor progression (Brisson et al., 2016).

## 4 Discussion

In this research effort, we proposed a novel ML based tool named 3Mint for the analysis of 3 types of-omics data. We have experimented 3Mint using miRNA and mRNA expression profiles, and methylation profiles obtained from TCGA for BRCA molecular subtype identification problem. 3Mint utilizes biological knowledge to create groups, perform scoring and construct a model based on the significant groups. The genes within the informative groups provide insight into the understanding of targeted genes with prominent CpGs and miRNAs. Groups are scored to detect significant relationships between miRNAs, CpGs, and genes. A novel contribution of 3Mint is the idea of grouping, an improvement over the more conventional methods which focus on gene lists. 3Mint utilizes 3-way relationships, giving different levels of impact to each-omic data towards understanding the cellular behavior of defined groups for BRCA molecular subtypes.

In previous sections, we presented the method itself in detail; showed the performance analysis of 3Mint; exposed the identified disease related novel groups with their associated genes, miRNAs, CpGs; and displayed the association of the detected biosignatures. High performance metrics of 3Mint over the top 10 cumulative groups (as shown in Table 1) imply that the tool can successfully classify BRCA molecular subtypes. As shown in Supplementary Table S3B, for the classification of the BRCA luminal and ER-negative subtypes, 3Mint discovered a number of statistically significant, biologically important groups. These groups had overlaps with many different features (including miRNAs, CpGs, and genes), where these relationships were investigated from a number of different viewpoints, and presented in Supplementary Tables S5–S7, and in Figures 3–6. In Subsections 3.5–3.10, the possible roles of the identified genes, miRNAs, CpGs for BRCA development, and for the distinction of Luminal and ER-negative groups of BRCA are assessed with respect to the literature. As explained in Section 3.10, an interesting connection with the previous literature was the role of the miR-17/92 cluster, which appeared at several points in our 3Mint analysis. We also would like to note that as well as focusing on the genes and miRNAs, the interpretation of methylation data is also shown to be informative to understand differences between the defined BRCA molecular subtypes, as seen in Figures 5, 8, Supplementary Table S6 and as presented in Sections 3.6, 3.8, 3.10 Additionally, the biological relevance of the top scoring miRNA-CpG groups in terms of BRCA molecular subtype characterization is elaborated. Through several examples, we showed that the 3Mint tool could help to identify potential biomarkers for disease under investigation. The relationships between the cumulative groups, and between the mRNA, miRNA, and methylation markers provide insight into understanding of the basis of disease, mechanism of action and detection of disease state.

## 5 Conclusion

In this study, we developed 3Mint to elucidate the molecular mechanisms of heterogeneous diseases through exploring the interactions of features that are presented in multi-omics datasets. The proposed method enables grouping and scoring of features based on ML to improve the performance and interpretability of models *via* integrating gene expression data. Thus, the 3Mint tool enhances the comprehensive view of cellular signaling; and provides system level unveiling of cellular behaviors through analysis of 3-omics datasets. For future studies, we aim to add other-omics datasets such as genetic variants, transcription factors to improve the performance of the model. In addition, the proposed algorithm can be extended to more than 3-omics datasets (k-omics) to reach a more comprehensive knowledge about heterogenous disease mechanisms. Moreover, co-occurrence networks of multi-omics data with utilization of biological knowledge can be constructed to find central signatures and potential biomarkers for more precise diagnosis and for developing targeted therapies.

## Data Availability

The original contributions presented in the study are included in the article/Supplementary Material, further inquiries can be directed to the corresponding author.
