# Supplementary material for: Invention of 3Mint for feature grouping and scoring in multi-omics
Source: Front Genet. 2023 Mar 15;14:1093326. doi: 10.3389/fgene.2023.1093326 (PMC10050723; doi:10.3389/fgene.2023.1093326)
Supplement: Supplementary file 2 [file Table1.DOCX]

Supplementary Tables:

Supplementary Table S1. Comparative performance evaluation of different classifiers used within 3Mint for discriminating Luminal vs ER-negative classes, over 10- fold Monte Carlo cross-validation. For each classifier, the performance metrics obtained for 10 best scored groups are presented.

| Model | Accuracy | Precision | Specificity | Sensitivity | Recall | F-measure | Area Under Curve |
| --- | --- | --- | --- | --- | --- | --- | --- |
| RF | 0.91 ± 0.05 | 0.91 ± 0.06 | 0.81 ± 0.15 | 0.97 ± 0.02 | 0.97 ± 0.02 | 0.94 ± 0.03 | 0.97 ± 0.02 |
| PNN | 0.88 ± 0.03 | 0.93 ± 0.06 | 0.87 ± 0.12 | 0.88 ± 0.04 | 0.88 ± 0.04 | 0.9 ± 0.02 | 0.95 ± 0.04 |
| NB | 0.93 ± 0.05 | 0.93 ± 0.07 | 0.85 ± 0.15 | 0.97 ± 0.02 | 0.97 ± 0.02 | 0.95 ± 0.04 | 0.95 ± 0.04 |
| GBT | 0.93 ± 0.05 | 0.93 ± 0.07 | 0.85 ± 0.15 | 0.97 ± 0.02 | 0.97 ± 0.02 | 0.95 ± 0.04 | 0.98 ± 0.03 |

Supplementary Table S2. The comparative performance evaluation of 3Mint with single-omics data analysis (mRNA only) and two-omics data analysis (mRNA and miRNA data) for the classification of Luminal and ER-negative groups.

| Method (Using RF model for 10 fold MCCV) | Average Gene # | Accuracy | Sensitivity | Specificity | F-score | Area Under Curve |
| --- | --- | --- | --- | --- | --- | --- |
| 3Mint_top group_mRNA, miRNA, methylation | 5.2 | 0.90±0.06 | 0.97±0.03 | 0.75±0.15 | 0.93±0.04 | 0.87±0.09 |
| miRcorrNet_top group_mRNA, miRNA | 2.4 | 0.78±0.09 | 0.95±0.07 | 0.46±0.08 | 0.86±0.03 | 0.75±0.17 |
| 3Mint_top 10 cumulative groups_mRNA, miRNA, methylation | 13.6 | 0.95±0.05 | 0.99±0.02 | 0.88±0.12 | 0.97±0.03 | 0.98±0.02 |
| miRcorrNet_top 10 cumulative groups_mRNA_miRNA | 38.2 | 0.94±0.04 | 0.97±0.03 | 0.89±0.1 | 0.96±0.03 | 0.99±0.01 |
| mRNA | 21839 | 0.99±0.01 | 0.99±0.01 | 0.98±0.02 | 0.99±0.01 | 1±0 |

Supplementary Table S3. 10 Most Significant Groups that are detected by 3Mint during the analysis of the BRCA molecular subtype datasets. The table summarizes frequency, average score and rank, number of associated genes and corresponding gene list. Minimum, median and maximum of the associated gene numbers over 100 splits are also included in the table.

| Group | Frequency of Group | Average Score | Average rank | # Associated Genes | Min Gene # for 100 splits | Median Gene # for 100 splits | Max Gene # for 100 splits | Associated Genes |
| --- | --- | --- | --- | --- | --- | --- | --- | --- |
| hsa-miR-20a_cg02370232 | 90 | 0.87 | 5.69 | 16 | 1 | 2 | 5 | BCL11A, HRK, SRSF12, L3MBTL4, … |
| hsa-miR-20a_cg06282596 | 88 | 0.88 | 5.35 | 14 | 1 | 2 | 6 | BCL11A, SRSF12, L3MBTL4, RNF8, …. |
| hsa-miR-17_cg06282596 | 47 | 0.88 | 5.72 | 17 | 1 | 4 | 8 | BCL11A, SRSF12, L3MBTL4, RNF8, … |
| hsa-miR-17_cg02370232 | 41 | 0.88 | 6.68 | 15 | 1 | 3 | 6 | BCL11A, SRSF12, L3MBTL4, DZIP1, .. |
| hsa-miR-19a_cg06282596 | 41 | 0.90 | 4.07 | 11 | 2 | 3 | 5 | RASAL1, BCL11A, SRSF12, AURKB, .. |
| hsa-miR-19a_cg02370232 | 40 | 0.90 | 3.95 | 8 | 2 | 3.5 | 5 | RASAL1, BCL11A, SRSF12, PIMREG, APBA2. |
| hsa-miR-20a_cg09791746 | 39 | 0.87 | 9.23 | 5 | 1 | 1 | 2 | BCL11A, SRSF12, RASAL1, LDHB, L3MBTL4 |
| hsa-miR-17_cg07212543 | 32 | 0.89 | 5.38 | 22 | 3 | 5 | 10 | ODC1, BCL11A, SRSF12, PIMREG, L3MBTL4,… |
| hsa-miR-20a_cg01909856 | 28 | 0.88 | 8.96 | 11 | 1 | 2 | 5 | BCL11A, SRSF12, L3MBTL4, ODC1, PIMREG… |
| hsa-miR-20a_cg24051242 | 19 | 0.89 | 8.11 | 18 | 1 | 6 | 9 | RNF8, BCL11A, CDKN2A, DZIP1, CDCA7, .. |

Supplementary Table S4. Functional enrichment analysis of the most frequently detected group (hsa-mir-20a_cg02370232)

| Category | Term | Count | % | P value | Genes | List Total |
| --- | --- | --- | --- | --- | --- | --- |
| GO_CC | GO:0005654~nucleoplasm | 7 | 0.5 | 0.025 | ZNF232, FOXC1, BCL11A, RNF8, PIMREG, AURKB, SRSF12 | 14 |
| GO_BP | GO:0051301~cell division | 3 | 0.214 | 0.026 | RNF8, PIMREG, AURKB | 14 |
| GO_MF | GO:0000978~RNA polymerase II core promoter proximal region sequence-specific DNA binding | 4 | 0.267 | 0.056 | ZNF232, FOXC1, BCL11A, OTX1 | 15 |
| GO_MF | GO:0003700~transcription factor activity, sequence-specific DNA binding | 3 | 0.2 | 0.062 | FOXC1, BCL11A,  OTX1 | 15 |

Supplementary Table S5. Top ten most significant miRNAs that are determined by 3Mint on BRCA molecular subtype datasets

| miRNA | Freq of miRNA | Total Freq in each group | Average Score | Average rank | # Associated Gene | # Associated CpGs | Associated Genes | Associated CpGs | Split list | Rank list |
| --- | --- | --- | --- | --- | --- | --- | --- | --- | --- | --- |
| hsa-miR-20a | 92 | 302 | 0.88 | 6.93 | 23 | 11 | RNF8, BCL11A, CDKN2A, DZIP1, … | cg24051242, cg01909856, cg06282596, cg02370232, … | 0, 0, 0, 0, 1, 1, 2, 2, 4, … | 1, 2, 3, 4, 5, 7, 1, 2, 1, …. |
| hsa-miR-17 | 56 | 202 | 0.89 | 6.49 | 36 | 17 | LDHB, RNF8, E2F3, BCL11A, …. | cg12427162, cg02370232, cg07212543,, … | 1, 1, 1, 1, 1, 1, 1, 5, 5, 5, 6, | 1, 2, 3, 3, 4, 4, 6, 5, 6, 9, 2, 4, … |
| hsa-miR-19a | 50 | 119 | 0.90 | 4.92 | 20 | 9 | RASAL1, BCL11A, CDCA7, RPIA, … | cg24051242, cg02370232, cg06282596, cg09791746, … | 5, 5, 5, 5, 6, 7, 7, 7, 7, 8, 9, 9, 12, | 1, 2, 3, 4, 1, 1, 2, 2, 13, .. |
| hsa-miR-92a-1 | 20 | 26 | 0.87 | 9.27 | 8 | 3 | BCL11A, RNF8, L3MBTL4, NRTN, .. | cg09791746, cg02370232, cg06282596… | 4, 5, 11, 13, 16, 24, 27, 30… | 7, 11, 3, 10, 8, 3, 2, 5, 9, … |
| hsa-miR-92a-2 | 15 | 15 | 0.86 | 10.27 | 3 | 1 | LDHB, BCL11A, L3MBTL4 | cg09791746… | 5, 7, 11, 13, 30, 34, 60, 68, 70, … | 7, 15, 1, 9, 10, 6, 3, 16, …. |
| hsa-miR-18a | 11 | 85 | 0.91 | 10.64 | 44 | 29 | IFRD1, CDC20, ATL2, BCL11A, RBM17, … | cg25979244, cg27005847, cg24296761, … | 12, 12, 12, 12, 12, 12,.. | 1, 2, 3, 4, 5, 6, 7, 8, 8, 9, 10, 11, … |
| hsa-miR-106b | 3 | 4 | 0.92 | 2.00 | 25 | 3 | NDC80, ORC1, CDK2AP1, LDHB, E2F3, .. | cg26242687, cg24296761, cg11524039… | 32, 33, 80, 80 | 2, 1, 1, 4 |
| hsa-miR-135b | 3 | 25 | 0.89 | 14.80 | 7 | 23 | SFT2D2, MICALL1, ANKS6, LINC00511, … | cg09569850, cg07212543, cg12427162, … | 12, 39, 39, 39, 39, 39, 39, … | 26, 3, 5, 6, 7, 8, 9, 10, .. |
| hsa-miR-19b-1 | 3 | 3 | 0.88 | 14.67 | 3 | 1 | BCL11A, L3MBTL4, APBA2 | cg02370232 | 42, 93, 97 | 11, 25, 8 |
| hsa-miR-4772 | 3 | 3 | 0.71 | 8.33 | 8 | 2 | CTSS, CD38, SLAMF7, GZMH, TNFRSF8, .. | cg13096007, cg24995678 | 57, 79, 90 | 4, 1, 20 |

Supplementary Table S6. Top ten most significant CpGs that are identified using 3Mint on BRCA molecular subtype datasets

| CpG | Freq of CpG | Total Freq in each group | Average Score | Average rank | # Associated Gene | # Associated miRNAs | Associated Genes | Associated miRNAs | Rank list | Split list |
| --- | --- | --- | --- | --- | --- | --- | --- | --- | --- | --- |
| cg02370232 | 92 | 182 | 0.88 | 5.86 | 23 | 6 | BCL11A, SRSF12, HRK, … | hsa-miR-20a, hsa-miR-17, hsa-miR-19a,… | 4, 2, 7, 1, 6, 2, 9, … | 0, 1, 1, 2, 4, 5, 5, 5, … |
| cg06282596 | 91 | 178 | 0.88 | 5.28 | 24 | 5 | BCL11A, SRSF12, L3MBTL4, … | hsa-miR-20a, hsa-miR-17… | 3, 4, 5, 2, 5,, … | 0, 1, 1, 2, 4, 5, … |
| cg09791746 | 42 | 86 | 0.87 | 9.17 | 5 | 5 | BCL11A, SRSF12, RASAL1, … | hsa-miR-20a, hsa-miR-92a-1, hsa-miR-19a, … | 3, 7, 4, 5, 7, 8, 11, … | 4, 4, 5, 5, 5,… |
| cg07212543 | 35 | 58 | 0.89 | 5.84 | 31 | 5 | ODC1, BCL11A, SRSF12, PIMREG, … | hsa-miR-17, hsa-miR-20a, hsa-miR-135b, … | 3, 5, 8, 7, 6, 7,, … | 1, 7, 7, 10,… |
| cg24051242 | 35 | 59 | 0.90 | 6.05 | 45 | 5 | RNF8, BCL11A, CDKN2A, .… | hsa-miR-20a, hsa-miR-19a, hsa-miR-17, … | 1, 4, 1, 1, 3, 9,, … | 0, 4, 5, 7, 7, 7, 10… |
| cg01909856 | 31 | 43 | 0.89 | 9.47 | 22 | 5 | BCL11A, SRSF12, L3MBTL4, ODC1, … | hsa-miR-20a, hsa-miR-17, hsa-miR-19a, … | 2, 4, 2, 6, 7, 3, 11,2,… | 0, 1, 4, 7, 7, 10,, … |
| cg12427162 | 24 | 33 | 0.91 | 5.42 | 56 | 6 | LDHB, RNF8, E2F3, BCL11A, SRSF12, … | hsa-miR-17, hsa-miR-20a, hsa-miR-18a, … | 1, 1, 2, 12, 15, 1, 10,, … | 1, 4, 10, 12, 12, … |
| cg26242687 | 20 | 22 | 0.90 | 6.00 | 34 | 4 | LDHB, E2F3, BCL11A, CDCA7, SRSF12, … | hsa-miR-17, hsa-miR-20a, hsa-miR-18a, … | 3, 3, 4, 8, 1, 4, 2, 6,, … | 1, 4, 10, 12, 18, … |
| cg13975098 | 10 | 11 | 0.90 | 9.64 | 27 | 3 | IFRD1, CDC20, ATL2, BCL11A, … | hsa-miR-18a, hsa-miR-17, hsa-miR-135b | 10, 5, 20, 3, 12, 7, .. | 12, 23, 39, 42,,… |
| cg24296761 | 10 | 10 | 0.92 | 2.80 | 35 | 2 | IFRD1, CENPA, CDC20, ATL2,… | hsa-miR-18a, hsa-miR-106b | 3, 1, 1, 8, 3, … | 12, 33, 39, 42, 49, .. |

Supplementary Table S7. The most significant CpG sites in 3Mint for classification of Luminal and ER-negative groups, the genes mapped to these CpG sites and chromosomal locations

| Probe ID | RefGene Name | Chromosome | Map Information | Gene location | CpG island regions |
| --- | --- | --- | --- | --- | --- |
| cg02370232 | SORBS1 | chr10 | 95415608 | Body | Open Sea |
| cg06282596 | SORBS1 | chr10 | 95415722 | Body | Open Sea |
| cg09791746 | ADAMTSL5 | chr19 | 1510495 | Body | Island |
| cg07212543 | MCF2L | chr13 | 112959423 | Unknown | Open Sea |
| cg24051242 | LRP5 | chr11 | 68328670 | Body | Open Sea |
| cg01909856 | NOTCH1 | chr9 | 136543838 | Body | N_Shore |
| cg12427162 | SFT2D2 | chr1 | 168229250 | Body | S_Shelf |
| cg26242687 | LINC01276,FOXP4-AS1 | chr6 | 41503276 | Unknown | N_Shore |
| cg13975098 | RGS10 | chr10 | 119544123 | Body | Island |
| cg24296761 | ARHGEF1 | chr19 | 41904306 | Body | Island |
| cg25581330 | PDLIM3 | chr4 | 185513839 | Body | Open Sea |
| cg27658601 | ZNF750,TBCD | chr17 | 82836205 | Body, 5'UTR | S_Shelf |
| cg08452338 | WWTR1 | chr3 | 149554203 | Body | Open Sea |
| cg15646741 | C1orf132 | chr1 | 207848391 | Unknown | Open Sea |

Supplementary Table S8. Top 10 most significantly enriched KEGG pathways in BRCA molecular subtype identification analysis

| KEGG ID | KEGG pathway name | Observed gene count | Background gene count | Strength | False discovery rate | Matching proteins in the network (labels) |
| --- | --- | --- | --- | --- | --- | --- |
| hsa05219 | Bladder cancer | 7 | 41 | 1.05 | 0.0023 | MDM2,E2F3,TP53,CDKN2A,DAPK3,MYC,RPS6KA5 |
| hsa03010 | Ribosome | 11 | 130 | 0.75 | 0.0023 | RPL18A,RPS27A,RPS15A,RPL7,RPL21,RPSA,RPS27,MRPS6,RPL17,RPL30,RPS10 |
| hsa04919 | Thyroid hormone signaling pathway | 10 | 119 | 0.74 | 0.0025 | TSC2,KAT2A,MDM2,TP53,NOTCH1,PRKCB,MED16,NCOA3,MED12,MYC |
| hsa04115 | p53 signaling pathway | 6 | 72 | 0.74 | 0.0233 | TSC2,MDM2,TP53,RRM2,IGFBP3,CDKN2A |
| hsa05220 | Chronic myeloid leukemia | 6 | 75 | 0.72 | 0.0238 | MDM2,E2F3,TP53,SMAD4,CDKN2A,MYC |
| hsa00190 | Oxidative phosphorylation | 9 | 130 | 0.66 | 0.0104 | UQCRC1,ATP5B,SDHA,COX5A,MT-ATP6,MT-CO2,MT-ND4,MT-ND2,COX7B |
| hsa04110 | Cell cycle | 8 | 120 | 0.64 | 0.016 | MDM2,E2F3,TP53,SMAD4,PTTG1,MAD1L1,CDKN2A,MYC |
| hsa05206 | MicroRNAs in cancer | 10 | 160 | 0.61 | 0.0104 | MDM2,E2F3,TP53,NOTCH1,PRKCB,DNMT1,STMN1,CDKN2A,MYC,RPS6KA5 |
| hsa05224 | Breast cancer | 9 | 145 | 0.61 | 0.0148 | E2F3,FGF7,TP53,NOTCH1,LRP5,FZD9,NCOA3,BRCA2,MYC |
| hsa04218 | Cellular senescence | 9 | 150 | 0.6 | 0.016 | TSC2,MDM2,E2F3,TP53,PPP1CA,IGFBP3,ETS1,CDKN2A,MYC |
