# Supplementary material for: Invention of 3Mint for feature grouping and scoring in multi-omics
Source: Front Genet. 2023 Mar 15;14:1093326. doi: 10.3389/fgene.2023.1093326 (PMC10050723; doi:10.3389/fgene.2023.1093326)
Supplement: Supplementary file 3 [file Image1.pdf]

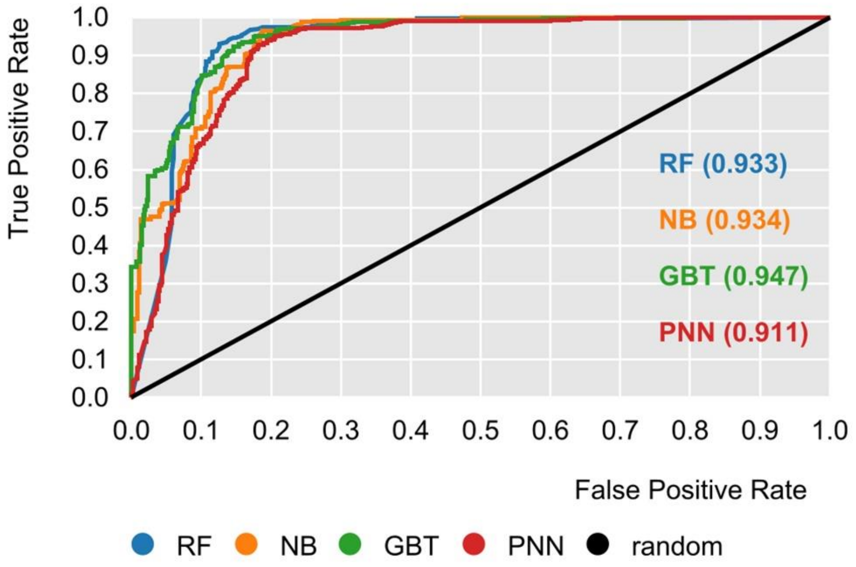

Supplementary Figure S1. ROC curves and AUC values of different models: Random Forest (RF), Naive Bayes (NB), Gradient Boosted Tree (GBT) and Probabilistic Neural Network (PNN)

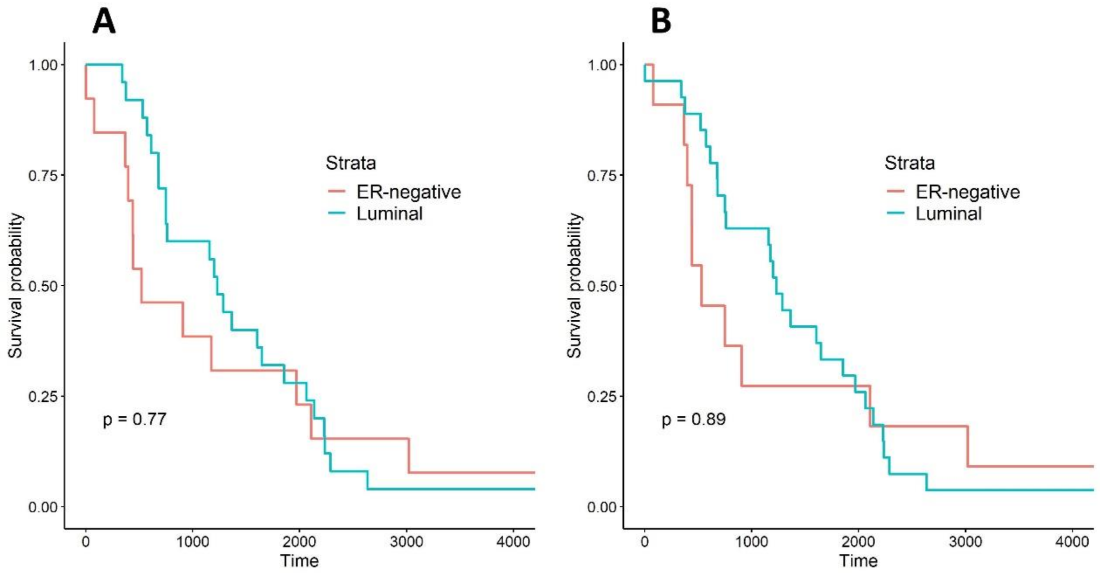

Supplementary Figure S2. Kaplan-Meier (K-M) survival plot for classification of ER-negative and Luminal groups, using (A) original class labels (B) predicted class labels obtained via Random Forest model in 3Mint.

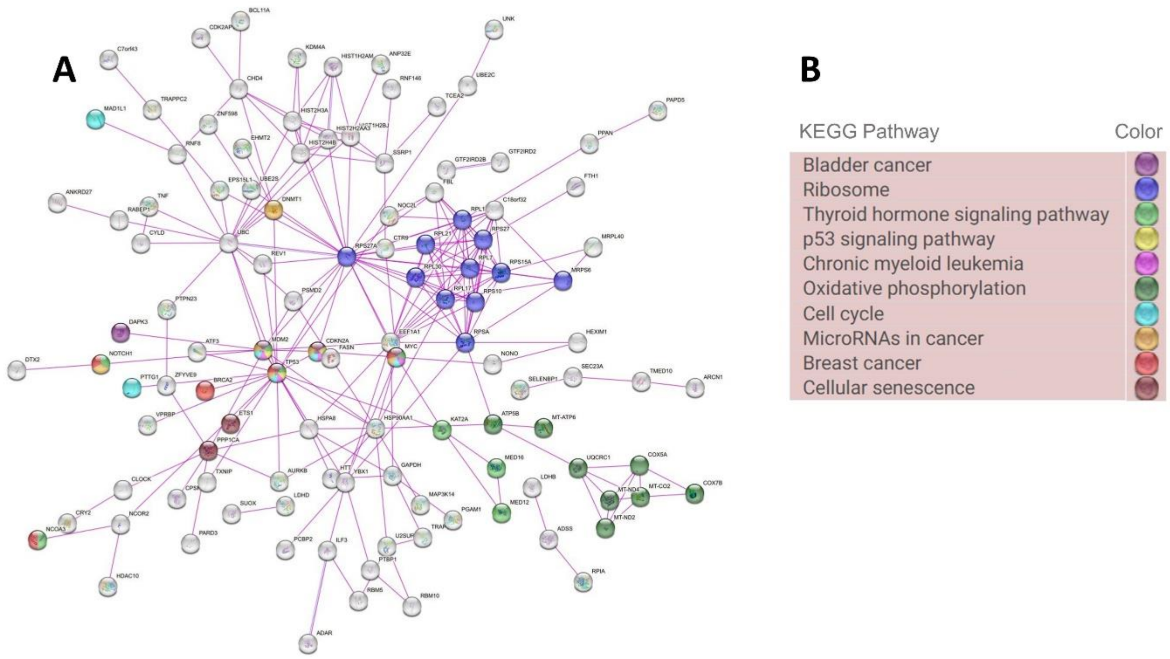

Supplementary Figure S3. Interaction network for BRCA Molecular Subtype Identification in 3Mint. (A) regulatory network for top 10 identified groups. (B) 10 most significant KEGG pathways (colors encoding functionally enriched pathways in the network)
